# A Plant Photoregulator‐Inspired S‐Type Heterojunction System for Diabetic Keratopathy via Tri‐Modal Light‐Driven Immunometabolic Reprogramming, Tissue Repair, and Antibacterial Activity

**DOI:** 10.1002/advs.76349

**Published:** 2026-07-01

**Authors:** Mengzhen Zhao, Zhibin Zhou, Yuxuan Wei, Xiangfan Huang, Zhiyong Zhou, Jiaheng Chen, Haoxin Cheng, Xiaotian Hu, Xiaolei Wang

**Affiliations:** ^1^ School of Chemistry and Chemical Engineering Nanchang University Nanchang Jiangxi P. R. China; ^2^ Department of General Surgery The First Affiliated Hospital of Nanchang University Nanchang Jiangxi P. R. China; ^3^ The National Engineering Research Center for Bioengineering Drugs and the Technologies Institute of Translational Medicine Nanchang University Nanchang Jiangxi P. R. China; ^4^ Center for Molecular Diagnosis and Precision Medicine The First Affiliated Hospital of Nanchang University Nanchang Jiangxi P. R. China

**Keywords:** catalytic cascade reaction, diabetic keratopathy, metabolic and immune regulation, photothermal antibacterial effect, tissue regeneration

## Abstract

Diabetic keratopathy (DK) is a prevalent ocular surface complication of diabetes, frequently unnoticed until significant structural and functional deterioration occurs. Chronic hyperglycemic stress promotes inflammation in the corneal epithelial‐neural‐immune (epineuroimmune) unit, impeding tissue recovery and increasing infection susceptibility. To address this metabolic–immune–infection imbalance, we developed a light‐regulated biomimetic catalytic platform (WCN_x_‐Rh2) that integrates glucose degradation and monitoring, immune‐modulated epithelial‐neural regeneration, and antibacterial defense. This platform features an S‐scheme heterojunction (WCN_x_) composed of graphitic carbon nitride and tungsten oxide, enabling visible‐light (VIS)‐driven glucose degradation, dark‐state colorimetric detection, and near‐infrared (NIR) photothermal antibacterial activity. Complementarily, the loaded ginsenoside Rh2—identified via systematic screening—inhibits the Nucleotide‐binding and oligomerization domain (NOD)‐like receptor signaling pathway. The combined action of WCN_x_ and Rh2 reduces advanced glycation end products (AGEs), reactive oxygen species (ROS), and inflammatory signaling, reprogramming dendritic cell function to restore epineuroimmune homeostasis and drive tissue repair. These functions were validated in two animal models. In diabetic mice, VIS‐irradiated WCN_x_‐Rh2 accelerated corneal epithelial and nerve recovery. In a diabetic keratitis model, NIR‐activated WCN_x_‐Rh2 enabled effective corneal bacterial eradication and tissue repair. Overall, this work establishes a light‐driven metabolic‐modulation biomimetic paradigm and proposes an integrated strategy for managing DK.

## Graphical abstract

To address the multifaceted imbalance in diabetic keratopathy, a light‐responsive biomimetic platform (WCN_x_‐Rh2) is developed and integrates glucose degradation, immune modulation, and antibacterial defense through a designed heterojunction and screened immunomodulator. WCN_x_‐Rh2 reduces AGEs/ROS and inflammatory signaling, reprogramming dendritic cells to restore epineuroimmune homeostasis. Both Diabetic and infected corneal models prove it as a novel metabolic‐immuno‐infectious regulatory strategy.

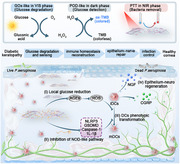

## 1 Introduction

Diabetic keratopathy (DK) is an important ocular surface complication of diabetes, affecting an estimated 47%–64% of diabetic patients [1, 2]. Clinically, DK is characterized by reduced corneal nerve density, delayed epithelial wound closure, increased susceptibility to microbial infection, corneal ulceration, and irreversible visual impairment in severe cases [3, 4]. A range of clinical therapies are available [5, 6, 7, 8], such as corticosteroid or non‐steroidal anti‐inflammatory eye drops to alleviate inflammation, neurotrophic factor therapy to promote epithelium–nerve repair, and topical antibiotics to control infection. However, these treatments fail to address the persistent dysregulation of glucose metabolism and immune activation that fundamentally drives disease progression. Moreover, reduced corneal sensitivity in diabetic patients delays symptom perception and diagnosis, thereby narrowing the therapeutic window and increasing the risk of severe corneal damage before clinical intervention [9]. Existing studies indicate that DK arises from dysregulation within the corneal epithelial‐neural‐immune (epineuroimmune) unit [10]. Therefore, an effective therapeutic strategy must simultaneously address four interrelated pathological dimensions: (1) Reduction and monitoring of local glucose levels. Chronic hyperglycemia induces the accumulation of advanced glycation end products (AGEs) and reactive oxygen species (ROS), which are initial triggers for chronic inflammation in the microenvironment [11]; however, effective local monitoring and intervention warning methods for sustained ocular hyperglycemia remain lacking. (2) Inhibition of dendritic cell (DC) immune activation. DCs are key resident immune cells within the epineuroimmune unit, and their maturation pathways are upregulated by AGEs and ROS, amplifying inflammatory responses [12, 13]. (3) Repair of epithelium and nerves. The vicious cycle of DC maturation and chronic inflammation reduces the availability of epithelial/neurotrophic factors, hindering epithelial proliferation and nerve regeneration, ultimately disrupting the unit homeostasis [14, 15, 16]. (4) Elimination of microbial infection. Structural and functional compromise of the epithelium and nerves renders diabetic corneas highly susceptible to opportunistic infections, further exacerbating tissue damage and delaying repair [17, 18].

Given these challenges, an ideal therapeutic strategy should achieve local glucose degradation with feedback monitoring, immune homeostasis reconstruction, epithelium–nerve repair, and infection control, thereby fundamentally restoring the structural and functional integrity of the diabetic cornea. In recent years, immunoregulatory biomaterials based on photo‐responsive materials and bioactive herbal monomers have demonstrated new potential in modulating pathological microenvironments [19, 20, 21, 22], which provide a feasible modular design framework for our present system: (1) a visible‐light (VIS)‐responsive cascade catalytic module that enables spatiotemporally controlled glucose degradation and detection via photoactivated glucose oxidase (GOx)‐ and peroxidase (POD)‐like cascades [23, 24, 25, 26]; (2) a bioactive herbal monomer module that offers multidimensional immune regulation, supported by reverse network pharmacology based on transcriptome sequencing and traditional Chinese medicine/compound molecular database for efficient identification of monomers [27, 28, 29, 30, 31]; (3) an immunomodulatory module that promotes tissue regeneration by restoring intercellular communication disrupted by chronic inflammatory imbalance [32, 33, 34, 35]; and (4) a near‐infrared (NIR) photothermal module that enables efficient, precise antibacterial action, providing an on‐demand light‐controlled mechanism for acute infection management [36, 37, 38, 39, 40]. Notably, this design is not a simple functional combination but a clinically oriented strategy that enables non‐invasive switching among metabolism and immune regulation for repairing, glucose monitoring for warning, and antimicrobial activity for defending to accommodate individualized DK treatment needs.

**SCHEME 1:**
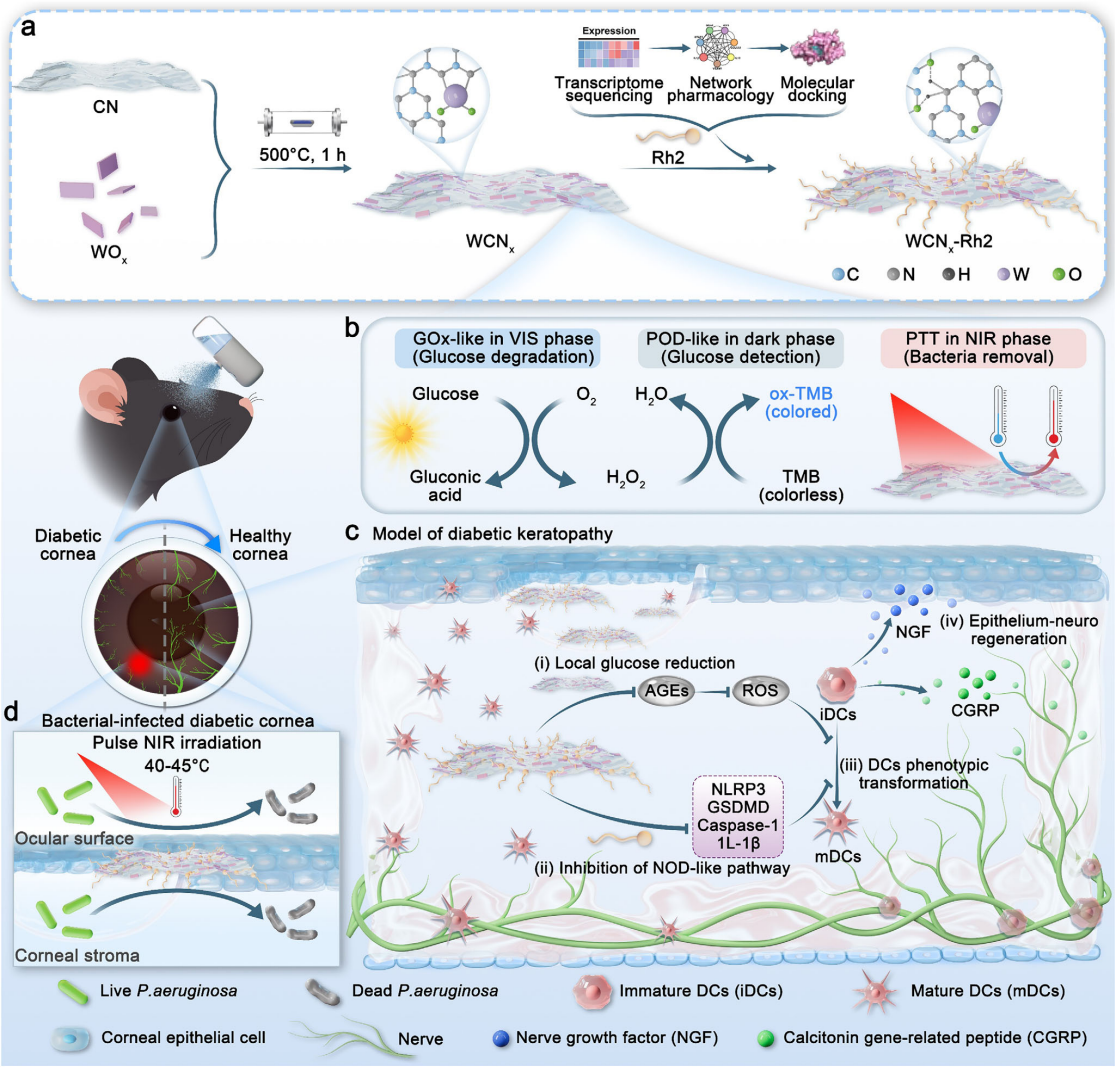
Schematic illustration of the preparation, light‐programmed functions, and therapeutic mechanisms of the WCN_x_‐Rh2 biomimetic catalytic system. (a) Synthetic diagram of WCN_x_‐Rh2, including the heterojunction fabrication and drug screening guided by reverse network pharmacology. (b) Three‐phase light‐controlled functions: VIS triggers GOx‐like glucose oxidation for degradation; darkness enables POD‐like H_2_O_2_‐driven TMB oxidation for colorimetric sensing; NIR induces photothermal heating for antibacterial treatment. (c) Proposed mechanisms of DK prevention by WCN_x_‐Rh2: i) local glucose reduction. By balancing ocular surface glucose levels, WCN_x_ suppresses the accumulation of AGEs and the generation of secondary ROS; ii) inhibition of NOD‐like pathway. Rh2 down‐regulates the expression of NOD‐like pathway by regulating the level of related factors; iii) DC phenotypic transition. Through the above two points of regulation, WCN_x_‐Rh2 inhibits DC maturation; iv) epithelial and neural tissue regeneration. WCN_x_‐Rh2 restores DC homeostasis to reinstate the supply of epithelial and neurotrophic factors, thereby protecting epithelium and nerves. (d) Photothermal therapy (PTT) of diabetic bacterial keratitis using WCN_x_‐Rh2.

Based on the above considerations, this study draws functional inspiration from how plants use light as a metabolic pacemaker, activating or silencing metabolism through VIS/dark cycles and enhancing microbial resistance under NIR irradiation [41]. Accordingly, a biomimetic catalytic platform (WCN_x_‐Rh2) was constructed, which exhibited a “VIS‐driven metabolism/dark‐state sensing/NIR defense” rhythm for correcting the dysregulated DK microenvironment. WCN_x_‐Rh2 consists of an S‐scheme heterojunction (WCN_x_) formed by graphitic carbon nitride (CN) and tungsten oxide (WO_x_), combined with ginsenoside Rh2 (Rh2) identified via transcriptome‐guided reverse network pharmacology (Scheme 1a). The platform enables functional switching under three illumination conditions: VIS‐activated GOx‐like activity for glucose degradation, dark‐state POD‐like activity for visualizing glucose‐derived hydrogen peroxide (H_2_O_2_), and NIR‐responsive photothermal antibacterial action (Scheme 1b). This system manages DK through four integrated mechanisms: (I) Spatiotemporal control of glucose degradation and sensing. Under VIS, WCN_x_ catalyzes in situ glucose degradation to suppress AGEs/ROS accumulation, while in the dark, it decomposes glucose‐derived H_2_O_2_ to oxidize colorless TMB into blue ox‐TMB, thus enabling light‐switchable glucose sensing. (II) DC phenotype reversal via AGEs/ROS/NLRP3 downregulation. Rh2‐mediated inhibition of NOD‐like receptor signaling, together with WCN_x_‐driven glucose control, collectively suppresses DC activation to restore immune homeostasis (Scheme 1c(i–ii)). (III) Immune‐balance for epithelium–nerve regeneration. Reversal from mature to immature DC phenotypes restores the supply of epithelial and neurotrophic growth factors, facilitating epithelium–nerve repair (Scheme 1c(iii–iv)). (IV) Photothermal treatment of corneal infection. Under infectious stress, NIR pulse irradiation elevates local temperature to 40°C–45°C, enabling controllable bacterial eradication without damaging corneal tissue (Scheme 1d). By integrating metabolic correction, immune reprogramming, and infection control within a light‐responsive framework, this multimodal‐responsive system provides a flexible modular therapeutic solution for comprehensive DK management.

## 2 Results and Discussion

### 2.1 Design and Characterization of the Biomimetic Catalytic Platform

**FIGURE 1:**
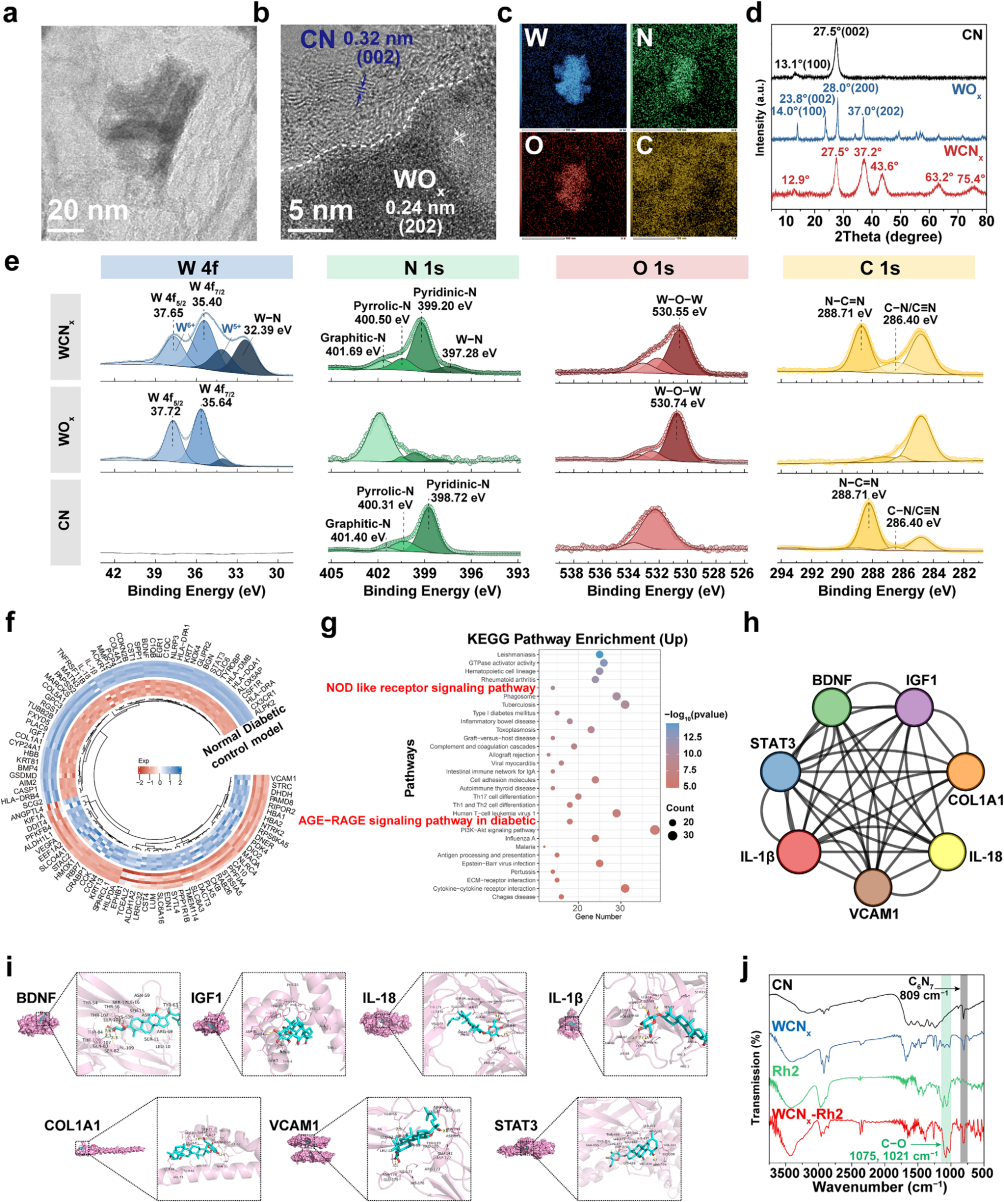
Characterization of the light‐controlled cascade catalyst and transcriptome‐guided drug screening. (a) TEM image, (b) HRTEM image, and (c) EDS mapping of WCN_x_. (d) XRD patterns of CN, WO_x_, and WCN_x_. (e) High‐resolution XPS spectra (W 4f, N 1s, O 1s, and C 1s) of CN, WO_x_, and WCN_x_. (f) Heatmap of DEGs between diabetic and normal mice obtained from bulk RNA sequencing (*n* = 6). (g) KEGG pathway enrichment analysis based on RNA sequencing data. (h) Identification of key genes from the PPI network using Cytoscape. (i) 3D molecular docking representation of Rh2 with key targets. (j) FTIR spectra of CN, WCN_x_, Rh2, and WCN_x_‐Rh2.

The synthesis of the WCN_x_ heterojunction using a sequential hydrothermal–calcination strategy. Following previously reported protocols, WO_x_ and CN were synthesized separately via solvothermal treatment and thermal condensation in air, respectively [42, 43]. The two components were uniformly mixed via ultrasonic dispersion, followed by solvent evaporation and calcination at 500°C for 1 h under argon to yield the final WCNx heterojunction. Transmission electron microscopy (TEM) image revealed that CN displayed a thin‐layer sheet‐like morphology with a smooth surface (Figure S1), while WO_x_ exhibited a stacked nanosheet with intact lattice fringes (Figure S2). After calcination, WO_x_ was anchored onto CN, and the WCN_x_ surface became rougher, indicating increased surface defects in CN and lattice defects in WO_x_ (Figure 1a). High‐resolution TEM (HRTEM) image showed two lattice spacings at the interface—0.32 and 0.24 nm—assigned to the (002) plane of CN and the (202) plane of WO_x_, respectively (Figure 1b) [44, 45]. Energy dispersive spectrometry (EDS) elemental mapping confirmed the presence of W, N, O, and C, confirming the coexistence of WO_x_ and CN domains within the heterojunction (Figure 1c). X‐ray diffraction (XRD) patterns showed sharp characteristic peaks for WCN_x_, with 12.9° and 27.5° corresponding to the (100) and (002) planes of CN, and 37.2° corresponding to the (202) plane of WO_x_, further confirming successful heterostructure construction (Figure 1d). Raman spectra further exhibited characteristic peaks of WO_x_ (O─W─O, 600–900 cm^−1^) and CN (C─N stretching, 1200–1550 cm^−1^), supporting the coexistence of both components within WCN_x_ (Figure S3) [46, 47]. The particle size of WCN_x_ measured by dynamic light scattering (DLS) was approximately 265.7 nm (Figure S4a). After ultrasonic dispersion, the absorption peak of WCN_x_ did not decrease significantly within 24 h of standing, and there was no obvious stratification in the solution (Figure S4b). This indicated that WCN_x_ had good stability in the solution and certain potential for biological applications.

X‐ray photoelectron spectroscopy (XPS) was used to analyze the chemical states of the samples (Figure 1e). In the W 4f spectra, WCN_x_ exhibited a lower W^6+^/W^5+^ ratio than WOx, indicating a reduction in W valence states. The peak at 32.39 eV further suggested the formation of W─N bonds [48]. In the N 1s spectra of WCN_x_, characteristic CN signals including pyridinic N (399.20 eV), pyrrolic N (400.50 eV), and graphitic N (401.69 eV) were retained, while a new peak at 397.28 eV corresponded to N─W bonding [49]. Together, the W 4f and N 1s analyses confirmed the formation of new interfacial chemical bonds rather than simple physical mixing, providing a pathway for interfacial electron transfer. O 1s spectra showed an increased peak near 533 eV in WCN_x_ compared with WO_x_, indicating reduced oxygen content [50]. In the C 1s spectra, binding energies at 286.40 eV and 288.71 eV in WCN_x_ were attributed to amino/cyano groups (C─N─H/C≡N) and sp^2^‐hybridized carbon (N─C═N), respectively [49]. Additionally, the W 4f_5/2_ (37.72 eV) and W 4f_7/2_ (35.64 eV) peaks of WO_x_ shifted to 37.65 and 35.40 eV in WCN_x_, whereas the N 1s peaks of CN (401.40, 400.31, and 398.72 eV) shifted upward to 401.69, 400.50, and 399.20 eV in WCN_x_. These collective shifts demonstrated electron transfer from CN to WO_x_ within the heterojunction.

During the progression of DK, persistent hyperglycemia serves as the initiating factor, while subsequent cellular dysfunction drives disease pathogenesis. Thus, beyond correcting the hyperglycemic microenvironment, rapid repair of damaged corneal tissue requires the identification of key molecular intervention targets. We employed a reverse network pharmacology approach to screen natural compounds, which was combined with glucose‐modulating strategies to construct a “metabolism–immunity” therapeutic system. To achieve this, we established a mouse model of DK and performed transcriptomic sequencing on corneal tissues from both diabetic and normal mice. Using |log2FC| > 1 and adjusted *p* < 0.05 as the screening threshold, 3563 differentially expressed genes (DEGs) were identified, of which 1372 were downregulated and 2191 were upregulated (Figure S5), with the top 100 DEGs shown in Figure 1f. Kyoto Encyclopedia of Genes and Genomes (KEGG) and Gene Ontology (GO) enrichment analyses revealed that the pathological mechanisms of DK might involve the NOD‐like receptor signaling pathway and the AGE–RAGE signaling pathway in diabetes (Figure 1g and Figure S6).

The 3563 DEGs were analyzed by STRING to construct a protein‐protein interaction (PPI) network, and key genes were identified using cytoNCA (Cytoscape). Two rounds of core gene screening were conducted using the median values as thresholds, resulting in seven PPI hub genes (Figure 1h and Figure S7). These seven genes were used as potential therapeutic targets for reverse prediction of active herbal components by the Traditional Chinese Medicine Systems Pharmacology Database and Analysis Platform (TCMSP), with bioavailability (≥ 30%) and drug‐likeness (≥ 0.18) as filtering criteria. Nine candidate compounds were identified: crocetin, Rh2, kaempferol, piperine, quercetin, fisetin, podophyllotoxin, aloe‐emodin, and irisolidone. Molecular docking was then performed to evaluate binding energy between these compounds and the PPI hub targets, revealing that Rh2 exhibited the lowest binding energies and highest affinities (Figure S8). Representative 3D docking conformations between Rh2 and the key DEGs are shown in Figure 1i, supporting its selection as the active molecular component for subsequent validation. Fourier‐transform infrared spectroscopy (FTIR) confirmed that WCN_x_‐Rh2 retained the ether ring signatures of Rh2 (1075 and 1017 cm^−1^) as well as the pyridine ring feature of CN (809 cm^−1^) (Figure 1j) [51]. Compared with the characteristic peak of the ether ring in Rh2 (1081 and 1022 cm^−1^), the corresponding characteristic peak of WCN_x_‐Rh2 shifted toward the lower wavenumber direction. This phenomenon indicated that the oxygen atom in the ether ring might combine with WCN_x_ through hydrogen bonds. A standard curve correlating Rh2 concentration with absorbance was established, and the drug‐loading capacity of bare WCN_x_ was determined to be 22.1% (Figure S9).

### 2.2 Light‐Controlled Cascade Catalytic Performance and Mechanism

We first investigated the optical properties of WCN_x_. Mott–Schottky plots (Figure S10) indicated conduction band (CB) potentials of −0.50, −0.62, and −0.34 V for WOx, CN, and WCNx, respectively. Based on the valence band spectra, the valence bands (VB) of WO_x_, CN, and WCN_x_ were 2.23, 1.90, and −0.28 eV, respectively (Figure S11). Therefore, the band gaps (Eg) of WO_x_, CN, and WCN_x_ were calculated as 2.73, 2.52, and 0.06 eV. These band‐structure features suggested a staggered band alignment, and its narrow Eg conferred enhanced conductivity and broader light absorption. Ultraviolet–visible–infrared (UV–vis–IR) spectra further showed stronger absorption of WCN_x_ in both VIS and NIR regions compared with CN and WO_x_ (Figure S12). Using a xenon lamp as the light source, we measured photocurrent responses and electrochemical impedance spectra (EIS), finding that WCN_x_ exhibited 4.5‐fold (vs. WO_x_) and 9‐fold (vs. CN) enhancements in transient photocurrents (Figure S13), and the smallest semicircle in the mid‐frequency region of EIS (Figure S14). These results demonstrated faster electron‐transfer kinetics and higher surface reactivity of WCN_x_. Efficient charge separation is crucial for photocatalysis, and the lower photoluminescence (PL) intensity of WCN_x_ relative to WO_x_ and CN indicated significantly suppressed recombination of photogenerated electron–hole pairs (Figure S15). Time‐resolved PL (TRPL) measurements further revealed prolonged carrier lifetimes: WCN_x_ displayed a lifetime of 8.57 ns at 441 nm, which is markedly longer than that of WO_x_ (3.94 ns) and CN (4.45 ns), indicating greatly inhibited charge recombination (Figure S16).

**FIGURE 2:**
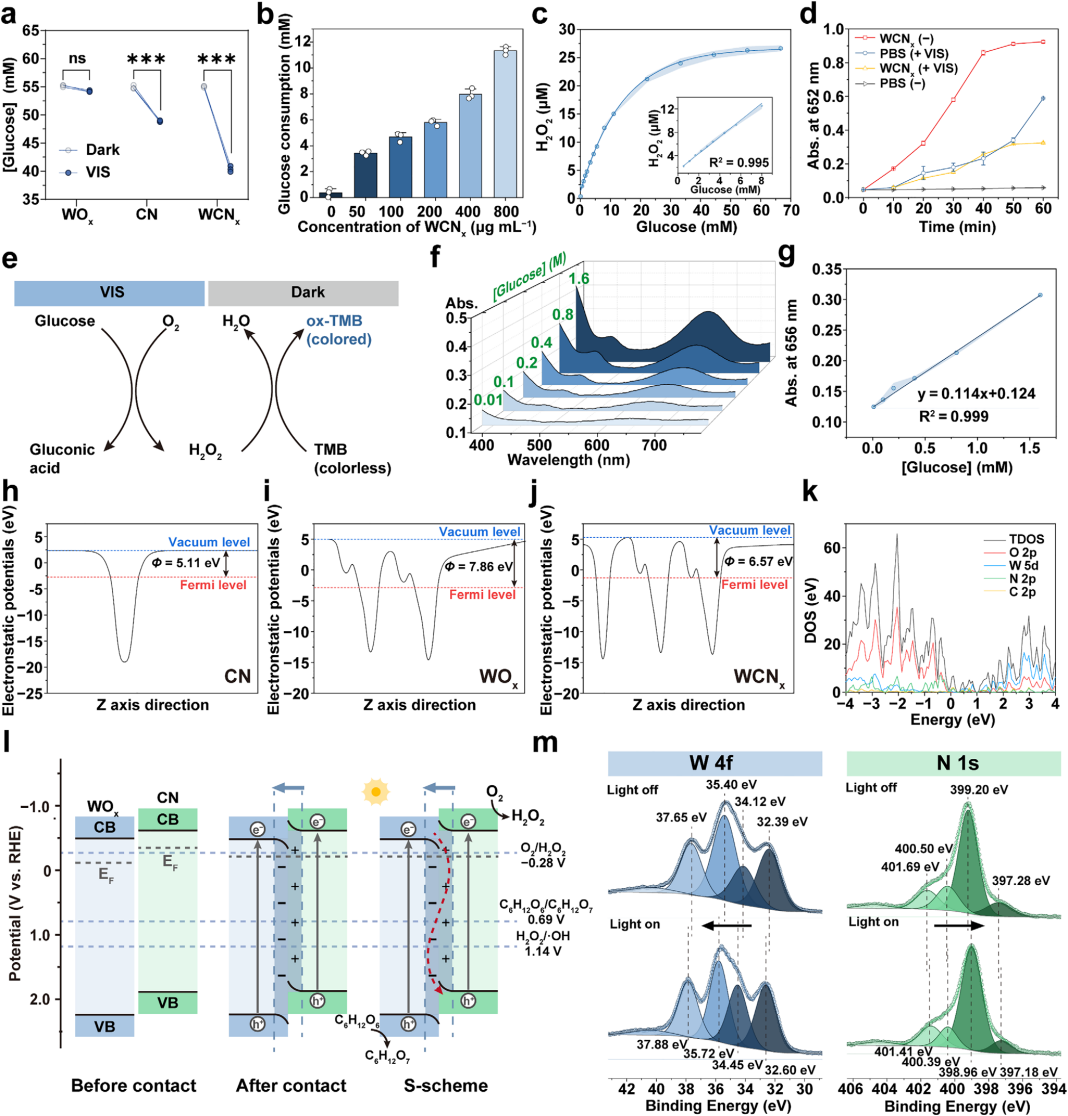
Light‐controlled cascade catalytic properties and mechanism of WCN_x_. (a) Glucose degradation by different materials with or without light irradiation. (b) Degradation of 50 mm glucose by WCN_x_ at different concentrations. (c) H_2_O_2_ generation corresponding to degradation of various glucose concentrations by 400 µg mL^−1^ WCN_x_. (d) Time‐dependent degradation of H_2_O_2_ by 400 µg mL^−1^ WCN_x_ under light and dark conditions. (e) Schematic illustration of the light‐controlled cascade catalytic process. (f) UV–vis spectra of TMB oxidation catalyzed in cascade reactions using different glucose concentrations as substrates. (g) Correlation between glucose concentration and TMB oxidation. (h) Electrostatic potential of CN, (i) WO_x_, and (j) WCN_x_. (k) DOS analysis of WCN_x_. (l) Band structure model and schematic of the photocatalytic reaction. (m) In situ XPS W 4f and N 1s spectra of WCN_x_. Data are means ± SD (*n* ≥ 3). ns = not significant, ***p < 0.001.

Based on its optical characteristics, we evaluated the glucose degradation capability of WCN_x_ using a xenon lamp emitting in the VIS range. We first compared the photocatalytic activities of WO_x_, CN, and WCN_x_ under light irradiation. As shown in Figure 2a, WCN_x_ consumed the largest amount of glucose, exhibiting a 2.5‐fold increase compared to CN and a 15‐fold increase compared to WO_x_, whereas negligible reaction occurred in the dark. Besides, WCN_x_ also showed concentration‐dependent catalytic behavior (Figure 2b). By incubating WCN_x_ with gradient concentrations of glucose under VIS for 30 min and subsequently quantifying the generated H_2_O_2_ using a detection kit, we found a strong correlation between H_2_O_2_ production and glucose concentration (Figure 2c). This result demonstrated that the H_2_O_2_ generated by WCN_x_ photocatalysis could serve as an indirect indicator of glucose concentration. We further evaluated the peroxidase‐like (POD‐like) activity of WCN_x_ using the TMB oxidation assay. Notably, WCN_x_ displayed catalytic activity toward TMB oxidation in the dark, whereas this activity was suppressed under VIS irradiation (Figure 2d). To examine the reaction kinetics, WCN_x_ was incubated with gradient concentrations of H_2_O_2_, and TMB oxidation was monitored at multiple time points. The reaction rate correlated linearly with substrate concentration and followed Michaelis–Menten kinetics characteristic of POD‐mimicking reactions (Figure S17).

Given the distinct catalytic activities under light and dark conditions, we hypothesized that VIS could serve as a switch to control a cascade reaction between glucose oxidation and TMB oxidation for indirect glucose detection. As shown in Figure 2e, WCN_x_ photodegraded glucose into H_2_O_2_ under VIS, and the in situ‐generated H_2_O_2_ was subsequently consumed in the dark to oxidize TMB. By adding TMB after illumination and incubating the reaction in the dark for 20 min, the accumulated H_2_O_2_ was converted into blue ox‐TMB via the POD‐like activity of WCN_x_. The ox‐TMB signal (absorbance at 656 nm) increased proportionally with glucose concentration (Figure 2f), showing a strong linear correlation within the range of 0–1.6 mM (Figure 2g), a range relevant for visualizing glucose variations in human tears (0.18–0.7 mm) [52]. These findings not only highlighted the cascade catalytic activity of WCN_x_ but also established VIS as the switching trigger controlling the two catalytic pathways, motivating further investigation into its catalytic mechanism.

To elucidate the photocatalytic mechanism of WCN_x_, density functional theory (DFT) calculations were performed. Work function analysis verified the direction of electron migration within the heterojunction. As shown in Figure 2h,i, the work functions of WO_x_ and CN were 7.86 and 5.11 eV, respectively, with their Fermi levels being −2.88 and −2.75 eV. Upon contact, electrons migrated from the component with lower work function to the higher one, generating an internal electric field (IEF) directed from CN to WO_x_ until Fermi level alignment was achieved (Figure 2j). Density of states (DOS) calculations further revealed the orbital contributions relevant to catalytic activity: O 2p and W 5d orbitals dominated the VB, while C 2p and N 2p orbitals dominated the CB (Figure 2k). The overlap between W 5d and N 2p orbitals near the Fermi level confirmed the formation of W–N bonds, enhancing electron transport and promoting photocatalytic reactivity.

Based on these results, we propose a potential photocatalytic mechanism for glucose degradation in the WCN_x_ heterojunction, where WO_x_ and CN function as oxidative photocatalyst and reductive photocatalyst, respectively. As illustrated in Figure 2l, electrons from CN (with a higher Fermi level) spontaneously diffuse into WO_x_ (with a lower Fermi level), establishing Fermi‐level equilibration and generating an internal IEF. This IEF facilitates charge separation, causing upward bending of the CN band edges due to electron depletion and downward bending of the WO_x_ band edges. Under illumination, the staggered band alignment forms an S‐scheme heterojunction interface. The built‐in IEF drives recombination between CB electrons of WO_x_ and VB holes of CN, retaining strong oxidative holes in the VB_WOx_ and strong reductive electrons in the CB_CN_. These carriers catalyzed glucose oxidation into gluconic acid and oxygen reduction into H_2_O_2_, respectively. In situ XPS further supports this mechanism: under illumination, W 4f peaks shifted positively and N 2p peaks shifted negatively in WCN_x_ (Figure 2m), confirming the S‐scheme electron transfer from CB_WOx_ to VB_CN_. Prior literature indicates that POD‐like catalytic activity in the dark arises primarily from surface defects [53, 54, 55]. Therefore, we used electron paramagnetic resonance (EPR) to analyze defect types, and Figure S18 shows significantly increased N and O vacancies in WCN_x_ compared with WO_x_ and CN, enabling more efficient H_2_O_2_ dissociation into reactive oxygen species, which subsequently oxidize TMB into blue ox‐TMB.

### 2.3 Suppression of AGEs/ROS and Phenotypic Reversion of DCs

**FIGURE 3:**
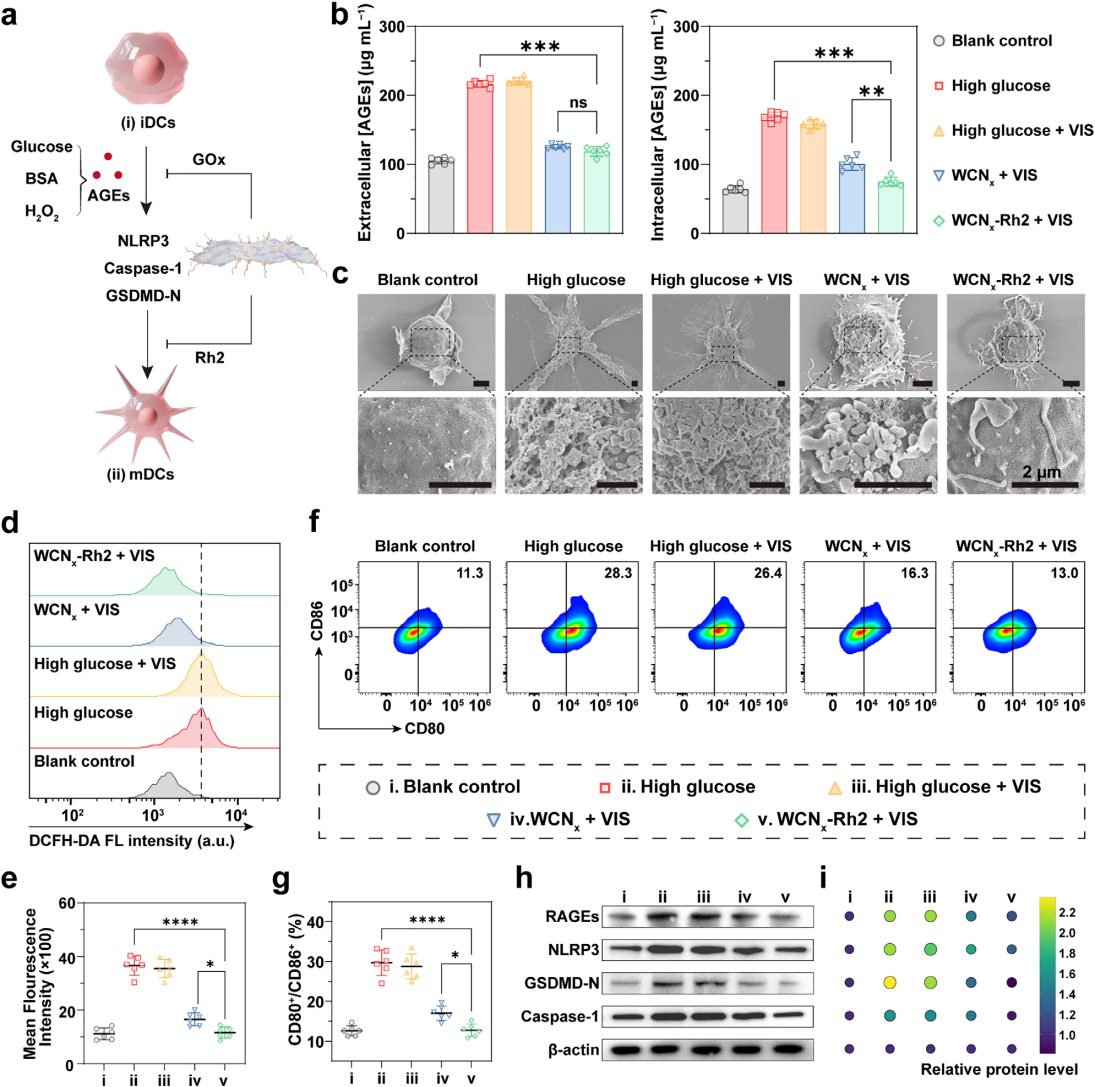
Regulation of DCs phenotype and underlying mechanisms. (a) Schematic illustration of the regulatory mechanism. (b) Inhibition of extracellular and intracellular AGE accumulation in DCs. (c) Electron microscopy images showing hyperglycemia‐induced morphological changes in DCs (mature DCs display dendritic, pro‐inflammatory morphology). (d) Flow cytometry analysis and (e) quantification of intracellular ROS induced by AGE accumulation. (f) Flow cytometry analysis and (g) quantification of phenotype reversal. (h) WB bands and (i) quantitative analysis of RAGE and NOD‐like signaling pathway‐related proteins. Data are means ± SD (*n* ≥ 3). ns = not significant, **p < 0.01, ***p < 0.001.

The accumulation of AGEs under hyperglycemia is the primary driver of microenvironmental dysregulation [11, 56]. DCs are the major resident immune cells in the cornea, of which functions were profoundly impaired in diabetic cornea [12, 13]. As shown in Figure 3a, AGEs‐induced oxidative stress imbalance caused DCs to transition from an immature state to a mature (pro‐inflammatory) phenotype. The aggravated inflammatory microenvironment further activates the NOD‐like signaling pathway, promoting continuous DC maturation and forming a vicious cycle of inflammation and immune stress. To prove this hypothesis, we investigated the effect of WCN_x_‐Rh2 on AGEs inhibition and AGE‐induced phenotypic alterations in DCs.

We first evaluated the biocompatibility of WCN_x_‐Rh2. Hemolysis assays showed that hemolysis rates remained below 5% across 25–800 µg mL^−1^ of WCNx‐Rh2 (Figure S19). CCK‐8 assays revealed no significant change in cell viability when WCN_x_ and WCN_x_‐Rh2 concentrations increased from 0 to 800 µg mL^−1^ (Figure S20), indicating negligible cytotoxicity and good biocompatibility toward DCs. Next, glucose, bovine serum albumin (BSA), and H_2_O_2_ were used to establish an in vitro hyperglycemia model to examine AGE inhibition. DCs were incubated for 24 h in media containing fixed BSA and various glucose/H_2_O_2_ combinations. Enzyme‐linked immunosorbent assay (ELISA) results showed that AGE levels positively correlated with glucose and H_2_O_2_ concentrations, and AGE accumulation was reduced in the WCN_x_ with VIS irradiation (Figure S21).

Based on these findings, we further investigated the effects of WCN_x_‐Rh2 under VIS on alleviating hyperglycemia‐induced stress in DCs. As shown in Figure 3b, both WCN_x_ and WCN_x_‐Rh2 reduced intracellular and extracellular AGEs formation under VIS irradiation. Scanning Electron Microscope (SEM) imaging showed that DCs in the high‐glucose model exhibited a mature, dendritic morphology, whereas cells in the WCN_x_ + VIS and WCN_x_‐Rh2 + VIS groups displayed an immature‐like morphology (Figure 3c). Intracellular ROS levels measured by DCFH‐DA demonstrated that WCN_x_‐Rh2 + VIS reduced ROS accumulation to levels comparable to normal cells, indicating effective alleviation of oxidative stress under hyperglycemia (Figure 3d,e). We then evaluated the expression of DC phenotypic markers. Under inflammatory or stress conditions, CD80 and CD86 are highly expressed on the surface as DCs undergo maturation. As shown in Figure 3f,g, the expression levels of CD80 and CD86 were markedly lower in the WCN_x_ + VIS and WCN_x_‐Rh2 + VIS groups than in the model group, indicating effective reversal of mature DCs back to an immature phenotype.

To further examine suppression of the NOD‐like signaling pathway, we performed Western blot (WB) analysis. RAGE, the cell‐surface receptor for AGEs, reflects the degree of glucose‐induced stress [57, 58]. Overactivation of NOD receptors can lead to inflammatory and degenerative processes. WB analysis showed that RAGE expression was significantly increased in the high‐glucose model, whereas VIS irradiation alone had minimal impact. In contrast, RAGE levels in the WCN_x_ + VIS and WCN_x_‐Rh2 + VIS groups were comparable to the blank control, indicating that local glucose suppression reduces RAGE expression. Notably, the WCN_x_‐Rh2 + VIS group also showed NLRP3, GSDMD‐N, and Caspase‐1 expression levels closest to the blank control (Figure 3h,i), demonstrating that combining Rh2 enabled more potent inhibition of the NOD‐like pathway. Collectively, these results indicated that WCN_x_‐Rh2 + VIS reduced local AGE accumulation and suppressed NOD‐like signaling activation, ultimately reversing hyperglycemia‐induced DC maturation and restoring an immature immune phenotype.

To further clarify the molecular basis of this reprogramming process, we additionally investigated STAT3‐related signaling. Docking analysis suggested a potential interaction between Rh2 and STAT3 (Figure S8). Previous studies have also linked STAT3 phosphorylation to DC maturation and connected the AGE/STAT3/NLRP3 axis with inflammatory activation under hyperglycemic conditions [59, 60, 61, 62]. Accordingly, we conducted WB analysis of STAT3 signaling and downstream inflammatory mediators. As shown in Figure S22, WCN_x_‐Rh2 + VIS exhibited the most pronounced inhibition of STAT3 phosphorylation, and this trend was accompanied by coordinated suppression of NOD‐like receptor‐related signaling molecules. Meanwhile, the downstream inflammatory end products IL‐18 and IL‐1β were also reduced most markedly in the WCN_x_‐Rh2 + VIS group. These findings suggest that, beyond relieving AGE/ROS‐associated upstream stress through WCN_x_‐mediated glucose catalytic regulation, Rh2 may further promote DC reprogramming by suppressing STAT3 phosphorylation and thereby weakening the propagation of NOD‐like receptor‐associated inflammatory signaling.

### 2.4 WCN_x_‐Rh2‐Mediated Regulation of Epithelium–Nerve Repair via DCs

**FIGURE 4:**
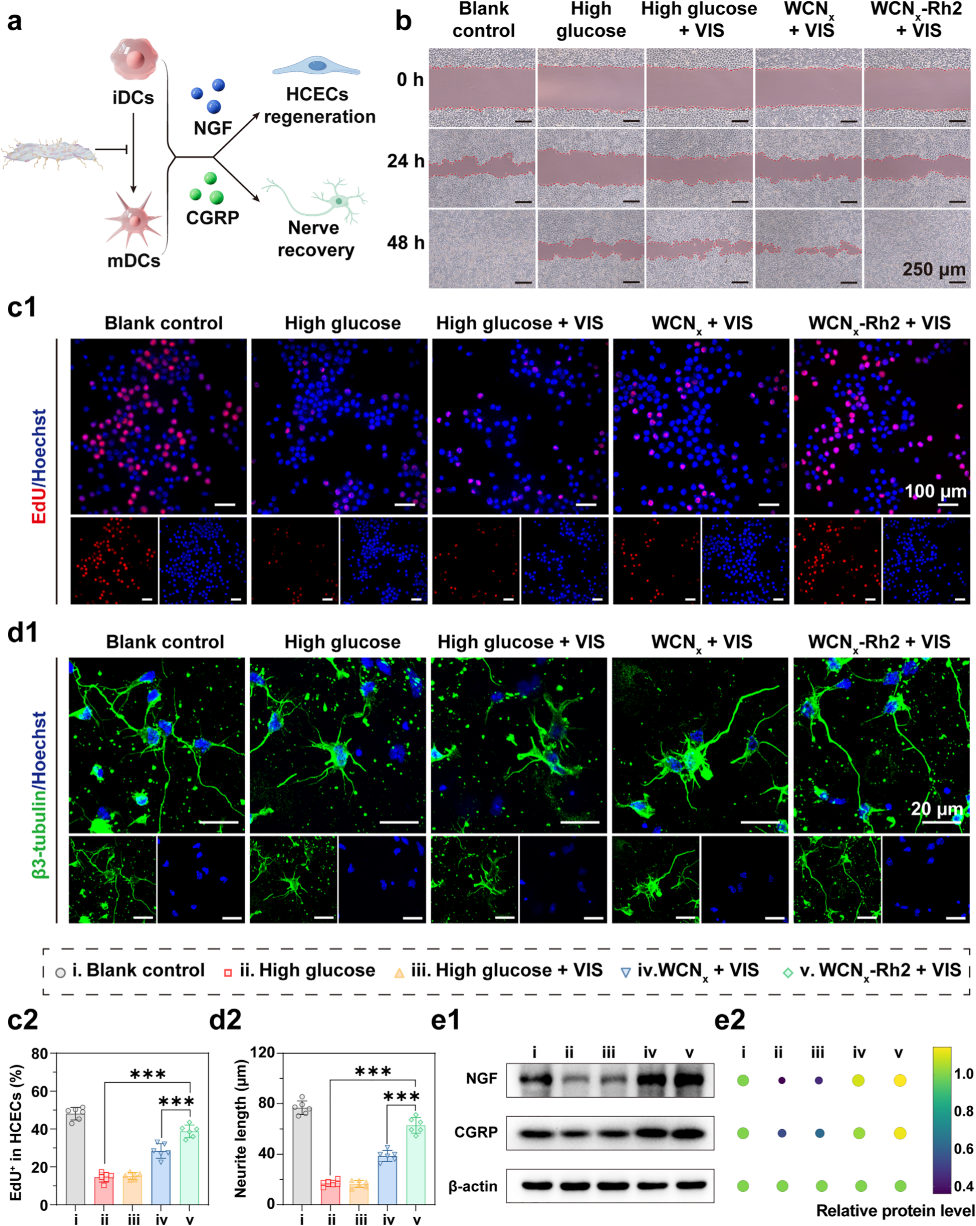
Interactions between DCs and epithelial/neuronal cells. (a) Schematic illustration. (b) Optical microscopy images of HCECs migration at different time points. (c1) EdU staining of HCECs proliferation and (c2) corresponding quantification. (d1) Neurite staining and (d2) corresponding quantification. (e1) WB bands and (e2) quantitative analysis of NGF and CGRP expression. Data are means ± SD (n ≥ 3). ***p < 0.001.

The corneal epineuroimmune unit, comprising epithelial, neuronal, and immune cells, constitute the major cellular components of corneal tissue and jointly maintain its microenvironmental homeostasis. During DC maturation, inflammatory cytokines released by activated DCs disrupt the physiological functions of corneal epithelial and neuronal cells, while simultaneously impairing the supply of nerve growth factor (NGF) and neuropeptides required for nerve regeneration, ultimately destabilizing corneal microenvironmental homeostasis (Figure 4a) [14, 15, 16]. Therefore, we conducted Transwell co‐culture experiments to further evaluate the role of inhibiting DC maturation through WCN_x_‐Rh2 in preserving epithelial and neuronal homeostasis.

We first assessed the influence of DCs on human corneal epithelial cells (HCECs). Wound healing assays and EdU staining were used to evaluate HCECs migration and proliferation after various treatments. DCs and HCECs were seeded in the upper and lower chambers, respectively. When HCECs reached 80–90% confluence, a scratch was created, and migration was recorded every 24 h. After 48 h, nearly 40% of the wound area remained unhealed in the high‐glucose model group, whereas the WCN_x_‐Rh2 + VIS group showed almost complete wound closure (Figure 4b and Figure S23). EdU staining further demonstrated that HCEC proliferation in the WCN_x_‐Rh2 + VIS group recovered to levels comparable to those of normal HCECs (blank control) (Figure 4c1,c2).

Using the same co‐culture system, we evaluated whether DC reprogramming could be translated into neuroprotective effects. β3‐tubulin staining showed that neurite outgrowth was markedly inhibited in the high‐glucose model group, whereas WCN_x_‐Rh2 + VIS largely preserved axonal extension (Figure 4d1,d2). This finding suggests that correction of DC dysfunction effectively relieved the inhibitory influence of the diabetic‐like inflammatory microenvironment on neuronal growth. Consistently, WB analysis showed that the neurotrophic factors NGF and CGRP in HCECs and neuronal cells were restored to near‐normal levels in the WCN_x_‐Rh2 + VIS group (Figure 4e1,e2). Thus, WCN_x_‐Rh2 + VIS not only protected neurite structure, but also improved the trophic microenvironment required for epithelial and neuronal maintenance. Together, these results support that reprogrammed DCs can alleviate neuroepithelial stress and contribute to restoration of epineuroimmune homeostasis, highlighting the nerve‐protective potential of this platform under diabetic conditions.

### 2.5 In Vitro Evaluation of Antibacterial Performance

**FIGURE 5:**
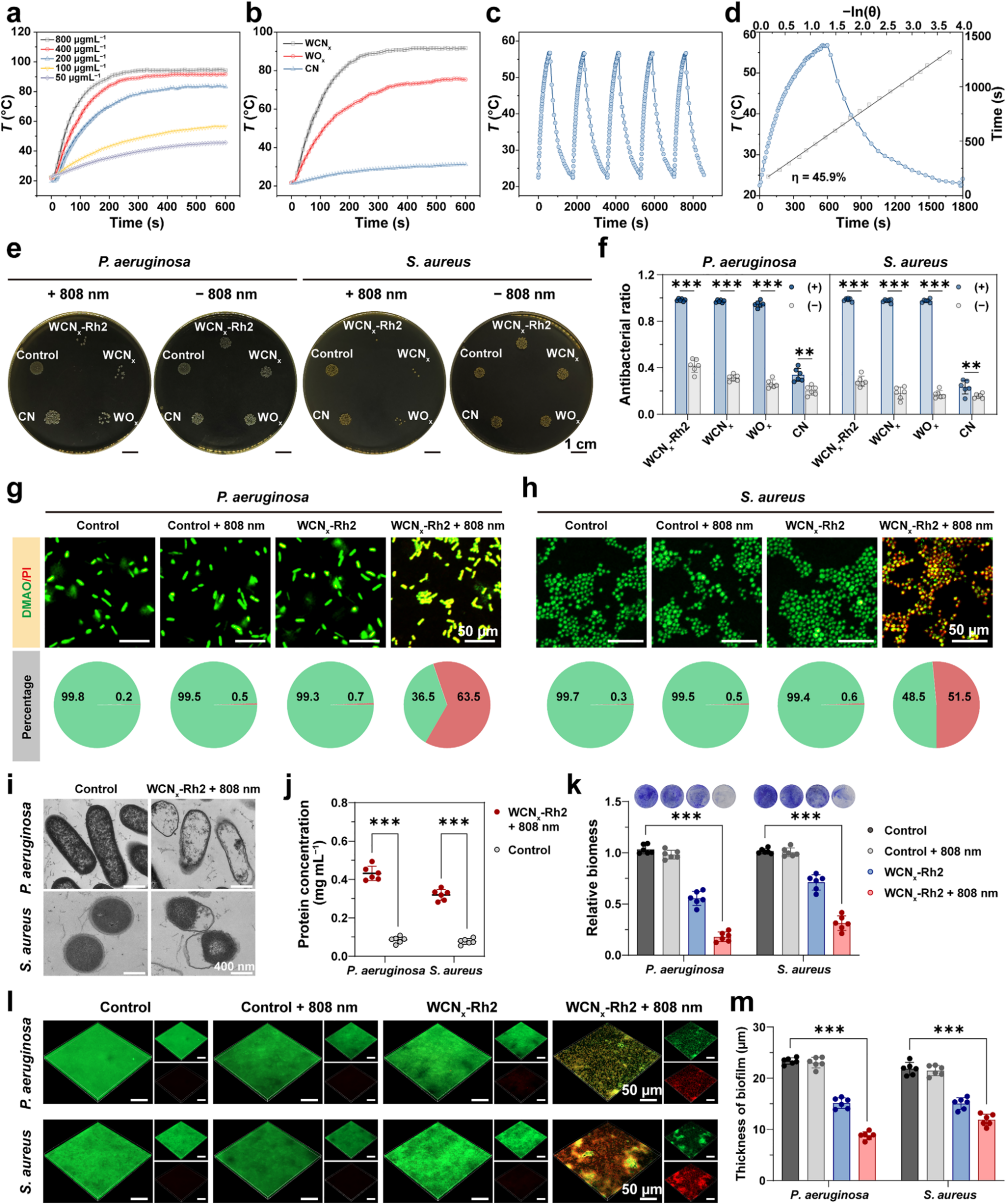
Evaluation of photothermal antibacterial activity and biofilm clearance. (a) Photothermal performance of WCN_x_ at different concentrations. (b) Photothermal performance of different component materials. (c) Heating profiles over five on/off irradiation cycles. (d) Photothermal conversion efficiency. (e) Antibacterial plate images and (f) quantitative antibacterial ratios of different materials with or without 808 nm irradiation. (g–h) Live/dead bacterial staining. (i) TEM images of bacteria with various treatments. (j) BCA assay of protein leakage. (k) Crystal violet staining and quantification of biofilms. (l) Confocal imaging of biofilms. (m) Quantification of biofilm thickness. Data are means ± SD (*n* ≥ 3). **p < 0.01, ***p < 0.001.

Microbial infection control is a critical component of managing DK, as diabetic patients not only exhibit increased susceptibility to opportunistic pathogens due to impaired epineuroimmune function but also experience markedly delayed healing once infection occurs. The persistent hyperglycemic microenvironment compromises barrier integrity, dampens innate immune defenses, and promotes excessive inflammation, resulting in more frequent and challenging corneal infections [63, 64, 65]. Therefore, effective antimicrobial intervention is essential for preventing secondary infection‐driven damage and improving overall therapeutic outcomes in DK. WO_x_ is known to be an efficient photothermal material, and UV–vis–IR spectra indicated that WCN_x_ exhibited enhanced NIR absorption. Therefore, we investigated the photothermal heating behavior of WCN_x_ under 808 nm irradiation and its corresponding antibacterial performance. WCNx exhibited concentration‐dependent photothermal heating (Figure 5a) and outperformed both precursor materials, enabling rapid temperature rising at 1 W cm^−^^2^ (Figure 5b,c). After five on/off heating cycles, WCN_x_ maintained stable photothermal performance, and the calculated photothermal conversion efficiency was 47.5% (Figure 5d). Based on these results, 1 W cm^−2^ was selected for subsequent antibacterial assays.

Taking *Pseudomonas aeruginosa* (*P. aeruginosa*) and *Staphylococcus aureus* (*S. aureus*) as models, we first evaluated the antibacterial effects of various material components and concentrations. After co‐incubating bacteria with different concentrations of materials for 6 h followed by 808 nm irradiation, plate counting showed that WCN_x_ achieved up to 99.8% antibacterial efficiency at 400 µg mL^−1^ (Figure S24). Using the same concentration (400 µg mL^−1^), WCN_x_ and WO_x_ exhibited stronger antibacterial activity than CN (Figure 5e,f). Live/dead bacterial staining demonstrated a significantly increased proportion of dead bacteria following WCN_x_‐Rh2 + 808 nm treatment (Figure 5g,h). TEM images further confirmed severe structural damage: *P. aeruginosa* displayed extensive cytoplasmic leakage, whereas *S. aureus* exhibited markedly thinned membranes and cell walls, cytoplasmic separation, and membrane disruption (Figure 5i). The increased extracellular protein release, quantified by bicinchoninic acid (BCA) assay, resulted from the material‐induced damage to bacterial integrity, further corroborating its antibacterial mechanism (Figure 5j). We next evaluated the capability of biofilm clearance. Crystal violet staining showed that WCN_x_‐Rh2 + 808 nm treatment effectively removed biofilms formed by *P. aeruginosa* and *S. aureus* (Figure 5k). 3D confocal laser scanning microscopy (CLSM) imaging of biofilm‐embedded bacteria revealed that WCN_x_‐Rh2 under 808 nm irradiation efficiently killed bacteria within the biofilm and significantly reduced overall biofilm thickness (Figure 5l,m).

### 2.6 WCN_x_‐Rh2‐Promoted Corneal Epithelial and Nerve Regeneration in Diabetic Mice

**FIGURE 6:**
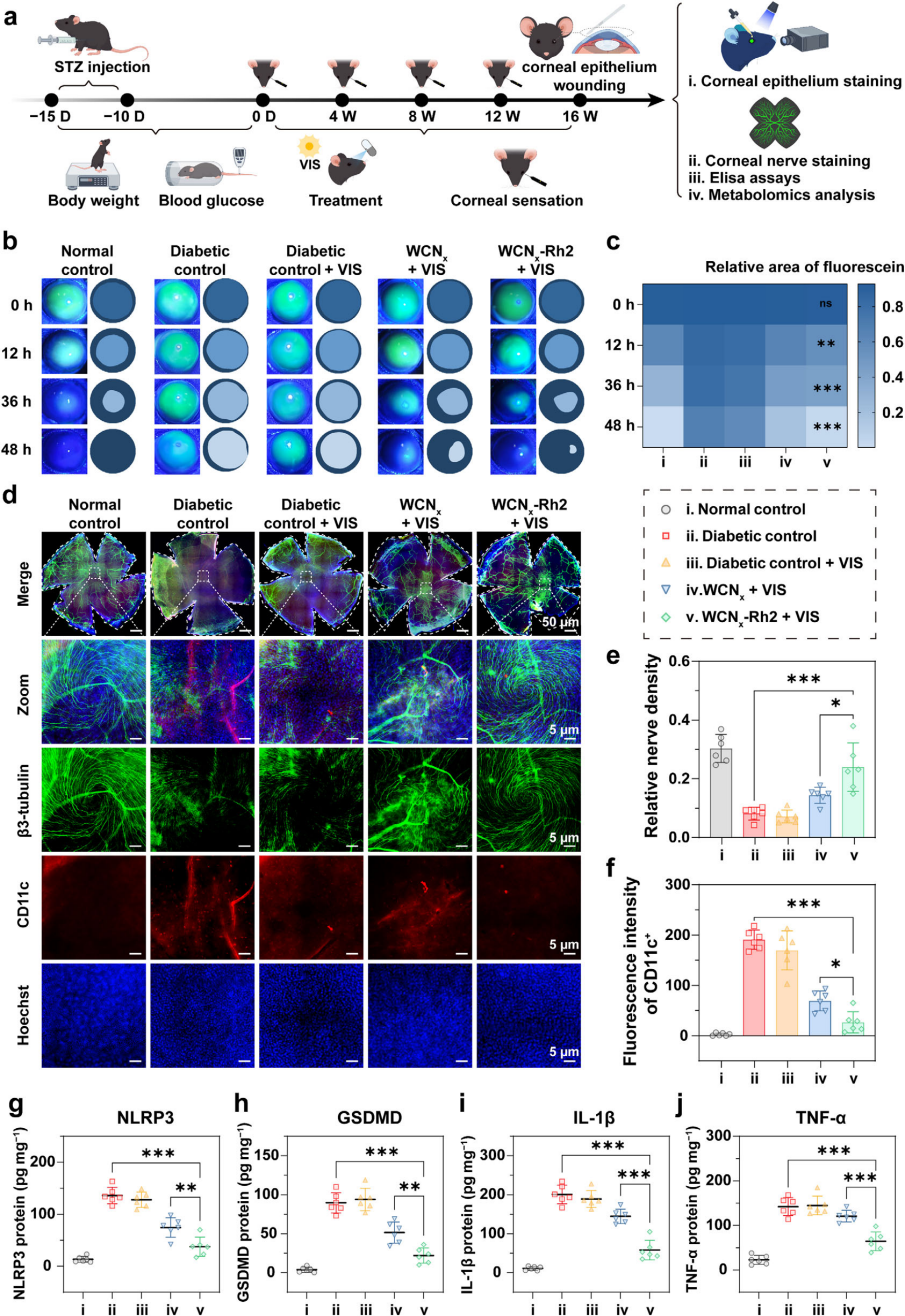
Evaluation of DK prevention. (a) Schematic diagram of the animal model. (b) Fluorescein staining for assessing corneal epithelial wound healing. (c) Quantification of wound closure area. (d) Fluorescence colocalization staining of corneal nerves and DCs. (e) Quantification of nerve density. (f) Quantification of CD11c^+^ in DCs. ELISA analysis of corneal samples showing levels of (g) NLRP3, (h) GSDMD, (i) IL‐1β, and (j) TNF‐α. Data are means ± SD (*n* = 6). ns = not significant, *p < 0.05, **p < 0.01, ***p < 0.001.

Delayed corneal epithelial repair and reduced corneal nerve density are hallmark manifestations of DK. To investigate the effects of WCN_x_‐Rh2 on corneal wound healing and nerve regeneration under pathophysiological conditions, a diabetic model was established in C57BL/6J mice. As shown in Figure 6a, after diabetes induction, diabetic mice were randomly assigned to four groups receiving twice‐daily treatment: diabetic control, diabetic control + VIS, WCN_x_ + VIS, and WCN_x_‐Rh2 + VIS, with an additional normal control group. To preliminarily assess the feasibility of local ocular‐surface action of WCN_x_‐Rh2 after topical instillation, Nile red‐labeled WCN_x_‐Rh2 was administered to the ocular surface of normal and DK mice, and the fluorescence distribution in ocular sections was observed at 0, 15, and 30 min (Figure S25). No obvious red fluorescence was detected at 0 min in either group, indicating a low background signal. Notably, fluorescence was already detectable in DK corneas at 15 min and became stronger at 30 min, whereas only a weak fluorescence signal was observed in normal corneas at 30 min. In both groups, the fluorescence was mainly localized to the ocular surface and the corneal epithelial/superficial corneal region. Considering the size effect of WCN_x_ and the lipophilicity of Rh2, we initially speculated that the WCN_x_ component was mainly present in the extracellular/local microenvironment, while Rh2, as a small‐molecule active ingredient, could enter cells through membrane‐related processes and participate in intracellular signal regulation [66, 67, 68, 69, 70, 71]. These preliminary findings supported the feasibility of WCN_x_‐Rh2 acting predominantly through a local ocular‐surface mode of action under DK conditions.

The neuroprotective effect of WCN_x_‐Rh2 + VIS was further validated in vivo. Corneal sensitivity testing performed every four weeks showed that diabetic mice exhibited a progressive decline in corneal sensation, whereas WCN_x_‐Rh2 + VIS treatment significantly improved corneal sensitivity compared with untreated diabetic controls (Figure S26). Because corneal sensitivity directly reflects the functional integrity of corneal innervation, this result indicates that the treatment not only alleviated local pathological stress, but also contributed to functional recovery of the diabetic cornea. Following 16 weeks of continuous treatment, a corneal epithelial debridement model was then established for further evaluation of wound closure by fluorescein staining. At 48 h post‐debridement, diabetic control and diabetic control + VIS groups showed only ∼20% wound closure, indicating markedly delayed healing, whereas the WCN_x_‐Rh2 + VIS group achieved ∼96% closure (Figure 6b,c). To further evaluate post‐treatment biosafety, H&E analyses were performed on ocular tissues and major organs. H&E staining showed no obvious pathological abnormalities in the cornea, iris, ciliary body, and crystalline lens after treatment (Figure S27). Meanwhile, histological examination of the heart, liver, spleen, lung, and kidney also revealed no evident tissue damage (Figure S28). These results indicated that WCN_x_‐Rh2 exhibited good local ocular safety and systemic in vivo biosafety.

To further examine the structural basis of this functional improvement, corneal flat mounts were prepared and co‐stained with β3‐tubulin to visualize nerve fibers and CD11c to label DCs. As shown in Figure 6d–f, compared with the normal control group, the diabetic control and diabetic control + VIS groups displayed markedly increased CD11c^+^ signals in the central cornea, accompanied by a pronounced reduction in central corneal nerve density. In contrast, WCN_x_‐Rh2 + VIS treatment reduced the abnormal accumulation of CD11c^+^ cells and significantly restored corneal nerve density. These findings are consistent with previous observations that corneal nerve loss during DK progression is closely associated with inflammatory DC activation and redistribution. ELISA was used to quantify inflammatory mediators and NOD‐related inflammatory factors in mouse corneal tissues. At 48 h post‐debridement, TNF‐α, IL‐1β, NLRP3, and GSDMD levels were markedly elevated in the diabetic control and diabetic control + VIS groups, indicating activation of the NOD pathway and substantial release of inflammatory cytokines. In contrast, the WCN_x_‐Rh2 + VIS group exhibited significantly lower expression of all these factors, demonstrating that WCN_x_‐Rh2 + VIS effectively suppressed NOD pathway activation and inflammatory cytokine production (Figure 6g–j). These in vivo results further support that WCN_x_‐Rh2 + VIS does not merely protect epithelial healing, but also improves the neuroimmune microenvironment required for corneal nerve maintenance and regeneration.

### 2.7 Metabolomics Analysis

**FIGURE 7:**
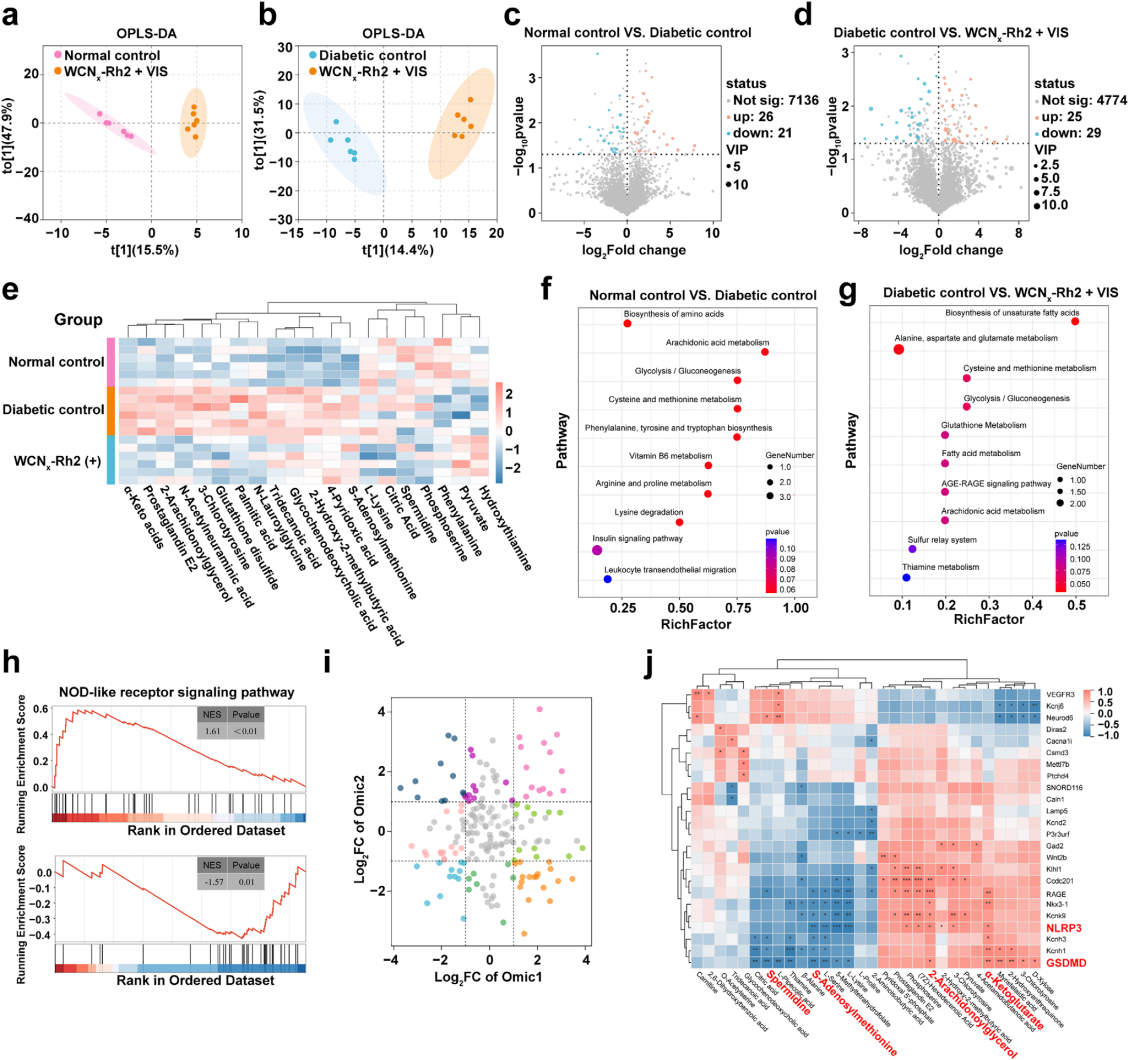
Multi‐omics integrated analysis of WCN_x_‐Rh2‐mediated corneal repair. (a, b) OPLS‐DA analysis of metabolomics data. (c) Volcano plot of differential metabolites between normal control and diabetic control groups, and (d) between diabetic control and WCN_x_‐Rh2 + VIS groups (red: upregulated metabolites, blue: downregulated metabolites). (e) Top 20 differential metabolites identified by metabolomics analysis. (f, g) Summary of the most enriched KEGG pathways, where bar length represents the number of metabolites involved and color gradient represents pathway significance (*p* < 0.05). (h) GSEA of RNA‐seq data for the NOD‐like receptor signaling pathway (top: normal control vs. diabetic control; bottom: diabetic control vs. WCN_x_‐Rh2 + VIS). (i) Nine‐quadrant plot illustrating transcriptome–metabolome correlation analysis. (j) Spearman correlation analysis of differential genes and metabolites. Data are means ± SD (*n* = 6). *p < 0.05, **p < 0.01, ***p < 0.001.

To validate the therapeutic efficacy of the material and elucidate its molecular mechanisms in the DK model, we performed integrated multi‐omics analyses. Metabolomics was first employed to provide key information on metabolic features under various physiological and pathological states. Data from liquid chromatography‐tandem mass spectrometry were subjected to multivariate statistical analyses, including principal components analysis (PCA) and orthogonal partial least‐squares discriminant analysis (OPLS‐DA), to evaluate metabolic differences among the normal control, diabetic control, and WCN_x_‐Rh2 + VIS groups. PCA results showed a clear separation between the normal control and diabetic control groups, whereas the WCN_x_‐Rh2 + VIS group clustered more closely with the normal control group, suggesting that metabolic alterations were associated with DK induction and WCN_x_‐Rh2‐mediated phototherapy (Figure S29). OPLS‐DA further confirmed robust intergroup separation (Figure 7a,b). Volcano plots comparing the three groups (Figure 7c,d) revealed 47 and 54 statistically significant metabolites (*p* < 0.05, VIP > 1.0), indicating that WCN_x_‐Rh2 + VIS treatment effectively modulated metabolic disturbances in diabetic corneas. As shown in Figure 7e, 20 enriched metabolites included lipids, organic acids, amino acids, and others. KEGG enrichment analysis based on differential metabolites indicated that pathways such as the tricarboxylic acid (TCA) cycle and glycolysis/gluconeogenesis predominated in the normal control vs. diabetic control comparison (Figure 7f,g). Notably, arachidonic acid metabolism and cysteine/methionine metabolism, two pathways associated with NOD‐like receptor activity, were also enriched, supporting their involvement in disease progression [72, 73, 74]. After treatment, enrichment analyses revealed that WCN_x_‐Rh2 + VIS modulated not only glucose‐related metabolic pathways but also arachidonic acid metabolism and cysteine/methionine metabolism. Furthermore, gene set enrichment analysis (GSEA) of RNA‐seq data demonstrated that the NOD‐like receptor signaling pathway was upregulated in the diabetic control group but reversed following WCN_x_‐Rh2 + VIS treatment (Figure 7h).

These findings suggest that arachidonic acid metabolism and methionine metabolism may play key roles in the therapeutic effects of WCN_x_‐Rh2 + VIS for DK. Integrative metabolomics–transcriptomics analysis is a powerful approach to uncover regulatory networks linking genotype to phenotype. To systematically explore the relationships between transcriptional and metabolic changes, a nine‐quadrant plot was used to illustrate the correlations between DEGs and differentially accumulated metabolites (DAMs) (Figure 7i). Quadrants 1, 2, and 4 represent upregulated genes accompanied by unchanged or downregulated metabolites. Conversely, quadrants 6, 8, and 9 represent downregulated genes with stable or increased metabolite levels. Quadrants 3 and 7 exhibit consistent trends between gene expression and metabolite accumulation, indicating potential positive regulation of metabolites by gene expression. Spearman correlation analysis between DAMs and DEGs was performed to further integrate the complex relationships between transcriptomic and metabolomic changes. As shown in Figure 7j, the correlation heatmap highlighted DAM–DEG pairs exhibiting strong associations (|PCC| > 0.8). Notably, the arachidonic acid metabolite 2‐arachidonoylglycerol showed strong positive correlations with key inflammation‐related genes such as NLRP3 and GSDMD, while methionine‐related metabolites S‐adenosylmethionine and spermidine exhibited negative correlations, consistent with previous reports [73, 75]. In addition, the TCA cycle intermediate α‐ketoglutarate showed positive correlations with NLRP3 inflammasome pathway, further indicating that WCN_x_‐Rh2 + VIS exerted integrated regulation over glucose metabolism, cellular energy metabolism, and tissue inflammation.

### 2.8 In Vivo Antibacterial Evaluation

**FIGURE 8:**
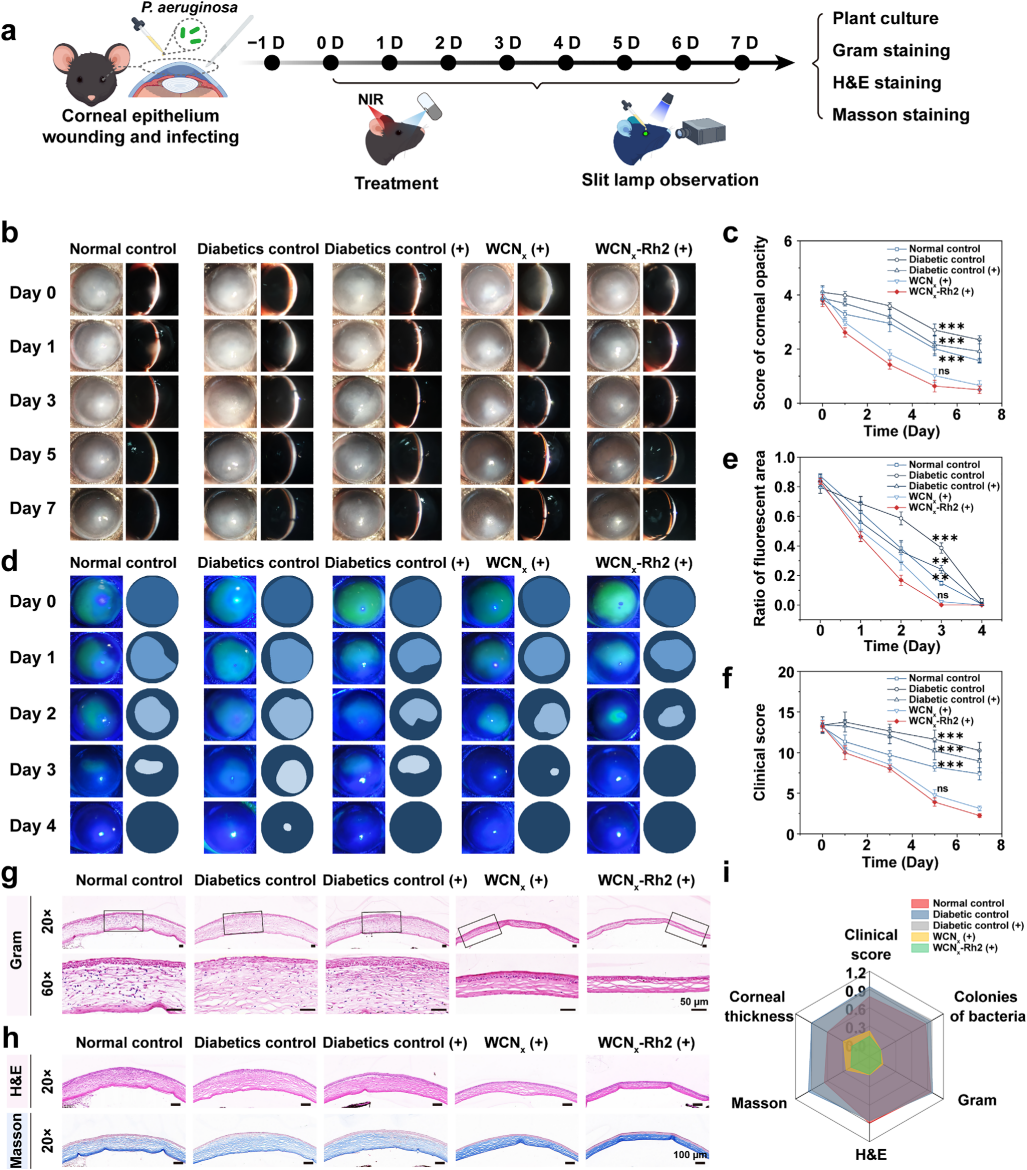
Therapeutic evaluation of bacterial keratitis in diabetic mice. (a) Schematic illustration of the animal model. (b) Optical images under diffuse illumination and slit lamp showing corneal opacity in each group. (c) Quantification of corneal opacity. (d) Fluorescein staining images assessing epithelial damage. (e) Quantification of epithelial defect area. (f) Clinical scores of keratitis. (g) Gram staining. (h) H&E and Masson staining. (i) Integrated normalized evaluation of multiple assessment outcomes. Data are means ± SD (*n* = 6). ns = not significant, **p < 0.01, ***p < 0.001.

To evaluate the in vivo antibacterial performance of the material, we established a *P. aeruginosa*‐induced diabetic keratitis model. As depicted in Figure 8a, a *P. aeruginosa*‐infected corneal wound was created one day before treatment initiation, followed by 7 consecutive days of intervention. The diabetic mice were randomly divided into four groups: diabetic control, diabetic control (+), WCNx (+), and WCNx‐Rh2 (+). The groups marked with (+) were irradiated with 808 nm light. Besides, an additional keratitis model without diabetics was set as the normal control group. As shown in Figure 8b, the control groups without materials treated exhibited reduced corneal transparency under diffuse illumination and slit‐lamp examination, indicating pronounced corneal edema. By day 5 of intervention, corneal transparency markedly improved in the WCN_x_‐Rh2 (+) and WCN_x_ (+) groups (Figure 8c). Fluorescein staining revealed that the WCN_x_‐Rh2 (+) group achieved complete wound closure by day 3, whereas the diabetic control group showed only ∼60% closure (Figure 8d,e).

Clinical corneal scoring showed that by day 7, the WCN_x_‐Rh2 (+) group scored around 2.5, indicating that inflammation had largely resolved (Figure 8f) [76, 77]. Ocular surface samples collected on days 0 and 7 were cultured on agar plates. Plate counting demonstrated that the WCN_x_‐Rh2 (+) and WCN_x_ (+) groups exhibited the lowest bacterial loads, confirming effective bacterial clearance from the ocular surface (Figure S30). Gram staining further revealed abundant *P. aeruginosa* infiltration in the stromal layer of the control group, while no obvious *P. aeruginosa* remained in the WCN_x_‐Rh2 (+) and WCN_x_ (+) groups (Figure 8g). These results collectively demonstrated that both WCN_x_‐Rh2 (+) and WCN_x_ (+) effectively eradicated bacteria from the ocular surface and stromal layers.

Hematoxylin and eosin (H&E) staining revealed extensive inflammatory cell infiltration in the normal control, diabetic control, and diabetic control (+) groups. In contrast, inflammatory infiltration was markedly reduced in the WCN_x_‐Rh2 (+) or WCN_x_ (+) groups, and the WCN_x_‐Rh2 (+) group exhibited a lower corneal thickness, suggesting minimal inflammatory response (Figure 8h). Masson staining was used to evaluate collagen deposition in the stromal layer. As shown in Figure 8h and Figure S31, the WCN_x_‐Rh2 (+) group displayed the densest and most organized collagen fibers, indicating superior stromal recovery. Collectively, these findings demonstrated that WCN_x_‐Rh2 (+) provided the most effective therapeutic outcome in vivo by integrating antibacterial activity, inflammation suppression, and stromal repair (Figure 8i).

## 3 Conclusion

In summary, we developed a tri‐modal light‐responsive biomimetic catalytic system (WCN_x_‐Rh2) with three major advantages: (I) More precise functional switching through light regulation. By integrating the distinct optical absorption properties and matched energy band structures of CN and WO_x_, WCN_x_ is capable of VIS‐driven glucose degradation, dark‐state H_2_O_2_‐catalyzed TMB oxidation, and NIR‐responsive photothermal antibacterial activity. Such multimodal, non‐destructive switching enables flexible, personalized intervention for diverse DK conditions. (II) Earlier metabolic warning through spatiotemporal catalytic switching. The dynamic transition between GOx‐like and POD‐like activities establishes a quantitative glucose–H_2_O_2_–TMB cascade, enabling intuitive visual readout of glucose fluctuations and providing an early metabolic warning signal within the ocular microenvironment. (III) Enhanced therapeutic integration through transcriptome‐guided molecular optimization. Reverse network pharmacology identified Rh2 as an immunomodulatory molecule capable of suppressing NOD pathway upregulation in DK corneas, supporting multidimensional immune regulation. Both in vitro and in vivo studies confirm that WCN_x_‐Rh2 enhances corneal epithelial and neural repair by regulating glucose‐related metabolism and reprogramming DC‐mediated immune activation. In the presence of infection, its NIR‐responsive photothermal effect enables efficient anti‐infective treatment and supports stromal recovery in the diabetic keratitis model. However, several limitations remain: (1) the efficiency of light‐controlled catalysis may vary with tear composition and individual physiological differences. (2) The loading stability and release kinetics of Rh2 lack precise controllability. (3) The long‐term biosafety of WCN_x_‐Rh2 and its potential immunological impacts require comprehensive evaluation. Taken together, the proposed biomimetic strategy, VIS‐driven metabolism modulation/dark‐state sensing/NIR defense, not only overcomes the limitations of single‐function DK therapies but also provides a flexible, customizable, multi‐modal light‐controlled therapeutic framework. In the future, this system holds promise for extension to other glucose dysregulation‐related conditions, including diabetic wounds, oral mucosal injuries, and regeneration of other epithelium–nerve composite tissues.

## 4 Methods

All the materials and methods can be found in the Supporting Information.

## Author Contributions

**Mengzhen Zhao**: conceptualization, methodology, investigation, validation, writing – original draft. **Yuxuan Wei**: validation. **Xiaolei Wang**: resources, supervision, funding acquisition, writing – review and editing, conceptualization, project administration. **Xiangfan Huang**: validation. **Haoxin Cheng**: validation. **Zhiyong Zhou**: validation. **Xiaotian Hu**: resources, funding acquisition, writing – review and editing. **Zhibin Zhou**: conceptualization, methodology, investigation, validation, writing – review and editing. **Jiaheng Chen**: validation.

## Conflicts of Interest

The authors declare no conflicts of interest.

## Supporting information




**Supporting File**: advs76349‐sup‐0001‐SuppMat.docx.

## Data Availability

The data that support the findings of this study are available from the corresponding author upon reasonable request.
