# Supplementary material for: A Plant Photoregulator‐Inspired S‐Type Heterojunction System for Diabetic Keratopathy via Tri‐Modal Light‐Driven Immunometabolic Reprogramming, Tissue Repair, and Antibacterial Activity
Source: Adv Sci (Weinh). 2026 Jul 1:e76349. Online ahead of print. doi: 10.1002/advs.76349 (PMC13336859; doi:10.1002/advs.76349)
Supplement: Supplementary file 1 — Supporting File: advs76349‐sup‐0001‐SuppMat.docx. [file ADVS-9999-e76349-s001.docx]

Supporting Information

**A Plant Photoregulator-Inspired S-Type Heterojunction System for Diabetic Keratopathy via Tri-Modal Light-Driven Immunometabolic Reprogramming, Tissue Repair and Antibacterial Activity**

*Mengzhen Zhao^†^, Zhibin Zhou^†^, Yuxuan Wei, Xiangfan Huang, Zhiyong Zhou, Jiaheng Chen, Haoxin Cheng, Xiaotian Hu*, and Xiaolei Wang****

M. Zhao*^†^*, J. Chen, H. Cheng, X. Hu***, X. Wang***

School of Chemistry and Chemical Engineering

Nanchang University

Nanchang, Jiangxi, 330031 (P.R. China)

Email: [happyhu@ncu.edu.cn](mailto:happyhu@ncu.edu.cn), [wangxiaolei@ncu.edu.cn](mailto:wangxiaolei@ncu.edu.cn)

Y. Wei, X. Huang, X. Wang***

The National Engineering Research Center for Bioengineering Drugs and the Technologies

Institute of Translational Medicine

Nanchang University

Nanchang, Jiangxi, 330088 (P.R. China)

Email: [wangxiaolei@ncu.edu.cn](mailto:wangxiaolei@ncu.edu.cn)

Z. Zhou*^†^*

Department of General Surgery

The First Affiliated Hospital of Nanchang University

Nanchang, Jiangxi, 330052 (P.R. China)

Z. Zhou

Center for Molecular Diagnosis and Precision Medicine

The First Affiliated Hospital of Nanchang University

Nanchang, Jiangxi, 330052 (P.R. China)

*†* These authors contributed equally to this work.

**1. Methods**

**1.1 Chemicals and Reagents**

Melamine (Aladdin, China), tungsten hexacarbonyl (W(CO)_6_, Macklin, China), ascorbic acid (AA, Macklin, China), oleylamine (OAm, Macklin, China), ginsenoside Rh2 (Rh2, Macklin, China), hydrogen peroxide (H_2_O_2_, Aladdin, China), 3,3′,5,5′-tetramethylbenzidine (TMB, Aladdin, China), and D-glucose (Macklin, China) were used as received without further purification. Phosphate-buffered saline (PBS) was purchased from Servicebio (China). Bovine serum albumin (BSA) was obtained from Biosharp (China). Dulbecco’s Modified Eagle Medium (DMEM), fetal bovine serum (FBS), and penicillin-streptomycin were purchased from Gibco (USA). Cell Counting Kit-8 (CCK-8) and EdU cell proliferation assay kits were obtained from Beyotime Biotechnology (China). The reactive oxygen probe 2′,7′-dichlorofluorescin diacetate (DCFH-DA) and the DMAO/PI bacterial viability staining kit were purchased from Beyotime Biotechnology (China). The BCA protein assay kit was obtained from Thermo Fisher Scientific (USA). Enzyme-linked immunosorbent assay (ELISA) kits for NLRP3, GSDMD, IL-1β, and TNF-α were purchased from Proteintech (China). Fluorescein sodium was obtained from Macklin (China). Paraformaldehyde (PFA) was provided by Servicebio (China). Proparacaine hydrochloride eye drops were obtained from Alcon (USA), and the anesthetic Sumianxin II (Shutai) was purchased from Southwest Pharmaceutical (China). Anti-β3-tubulin (657404) and Anti-CD11c (117307) antibodies were obtained from Biolegend (USA). Human corneal epithelial cells (HCECs), dendritic cells (DCs), and neuronal cells used in this study were obtained from the First Affiliated Hospital of Nanchang University (Nanchang, China). *Pseudomonas* *aeruginosa* (*P.* *aeruginosa*, ATCC 27853) and *Staphylococcus* *aureus* (*S.* *aureus*, ATCC 25923) were purchased from the American Type Culture Collection (ATCC, USA).

**1.2** **Synthesis of CN**

Melamine powder was directly placed in a covered alumina crucible and calcined in a muffle furnace under an air atmosphere. The temperature was increased from room temperature to 550℃ at a rate of 2–5 ℃ min^–1^, maintained for 4 h to complete the thermal condensation process, during which melamine polymerized and released ammonia gas to form graphitic carbon nitride. After natural cooling to room temperature, the resulting yellow solid was collected and ground into a fine powder to obtain bulk graphitic carbon nitride (CN). The product was then washed with deionized water and ethanol, followed by vacuum drying at 60℃ overnight for storage.

**1.3 Synthesis of WO_x_**

W(CO)_6_ (35 mg) and AA (20 mg) were introduced into a 15 mL vial containing OAm (5 mL). After sealing, the mixture was preheated in an oil bath at 40℃ for 30 min until the solution became clear. The reaction was then maintained at 250℃ for 5 h under static conditions. Upon natural cooling to room temperature, the obtained product was purified with an ethanol/cyclohexane mixture, followed by redispersion in cyclohexane for subsequent use.

**1.4 Synthesis of** **WCN_x_**

CN and WO_x_ were co-dispersed in ethanol, ultrasonicated for 30–60 min and stirred for 2–4 h to mix uniformly; the slurry was dried at 60–80℃, gently ground, loaded into a quartz boat, and calcined in a tube furnace under flowing argon (Ar) (≥ 100 mL min^–1^) by ramping 5 ℃ min^–1^ to 500℃ for 1 h. After natural cooling under Ar flow, the composite powder was re-dispersed in ethanol and ultrasonically exfoliated for 3 h to improve interfacial contact and lamellar dispersion, then collected by centrifugation, washed, and dried (60℃) to afford the WCN_x_ heterojunction.

**1.5 Synthesis of WCN_x_-Rh2**

The WCN_x_ powder was dispersed in 20–40% (v/v) ethanol/PBS (pH 7.4) at a concentration of 2.0 mg mL^–1^ by brief sonication (5 min). Rh2 was dissolved in ethanol (2 mg mL^–1^ stock solution) and added dropwise to the WCN_x_ dispersion at mass ratios (WCN_x_:Rh2) of 1:0.1–1:1. The mixture was gently shaken at 25℃ for 12 h to promote hydrogen bonding. The solid product was collected by centrifugation (12,000 rpm, 10 min), rinsed twice with 10% (v/v) ethanol/PBS to remove loosely bound Rh2, and vacuum-dried at 40–60℃ to obtain WCN_x_-Rh2. The amount of unbound Rh2 in the supernatant was quantified by UV–vis spectroscopy using a standard calibration curve to calculate the loading capacity.

**1.6 Instrumentation**

A transmission electron microscope (TEM, Zeiss, Germany) was employed to characterize morphology, lattice structure, and interfacial features. An X-ray diffractometer (XRD, Bruker, Germany) was used to determine crystal structures. An X-ray photoelectron spectrometer (XPS, Thermo Fisher Scientific, USA) was applied to analyze chemical states and valence-band spectra, as well as to evaluate interfacial electronic transfer between components. A Fourier-transform infrared spectrometer (FTIR, Nicolet, Thermo Fisher Scientific, USA) was used to identify functional groups and bonding configurations. An Ultraviolet–visible–infrared spectrophotometer (UV–vis–IR, Shimadzu, Japan) was employed to record optical absorption spectra, while a fluorescence spectrophotometer (Agilent Technologies, USA) and a transient/steady-state photoluminescence spectrometer (PL, Edinburgh Instruments, UK) were used to study charge-carrier recombination dynamics and photophysical properties. An electrochemical workstation (CH Instruments, China) was used to measure photocurrent responses and electrochemical characteristics. A microplate reader (Molecular Devices, USA) was used for optical density (OD) measurements. A flow cytometer (Sony Biotechnology, Japan) was employed to quantify dendritic cell maturation markers and intracellular reactive oxygen species (ROS) levels. A confocal laser scanning microscope (CLSM, Nikon A1, Japan), an inverted fluorescence microscope (Leica, Germany), and a digital slide scanner (Olympus, Japan) were used for fluorescence imaging of cells and tissues. A slit-lamp biomicroscope (Suzhou 66 Vision Technology, China) was used for in vivo observation of corneal opacity and epithelial wound healing. An SDS-PAGE electrophoresis system (Bio-Rad, USA) and a chemiluminescence imaging system (Bio-Rad, USA) were used for protein separation and Western blot detection. A liquid chromatography-tandem mass spectrometry system (LC–MS/MS, Thermo Fisher Scientific, USA) was employed for metabolomic profiling and quantitative metabolite analysis.

**1.7 RNA Sequence Analysis**

Total RNA extracted from corneas in the normal control and diabetic model groups was subjected to RNA sequencing analysis at OE Biotech Co., Ltd. (Shanghai, China). Differentially expressed genes (DEGs) between the two groups were identified using a threshold of |log2(fold change)| > 1 and adjusted *p*-value < 0.05, followed by hierarchical clustering visualization via a heatmap. Gene Ontology (GO) enrichment analysis was performed to categorize biological processes, molecular functions, and cellular components associated with the DEGs. Additionally, Kyoto Encyclopedia of Genes and Genomes (KEGG) pathway analysis was conducted to elucidate signaling pathways significantly enriched in the diabetic model group compared to the normal control group.

**1.8 Protein–Protein Interaction (PPI) Network and Natural Compound Screening**

A total of 3563 common targets were imported into the STRING database to construct a PPI network with a high confidence score (> 0.9). The resulting interaction network was exported and visualized using Cytoscape 3.10.1. The cytoNCA plugin was applied to calculate the Betweenness, Closeness, Degree, Eigenvector, local average connectivity (LAC), and Network parameters, while the CytoHubba plugin was used to identify the top seven hub genes within the PPI network and to construct the corresponding core subnetworks.

**1.9 Molecular Docking**

Molecular docking was employed to predict binding interactions between target proteins and candidate compounds. The 3D structures of target proteins were obtained from the Protein Data Bank (PDB) and meticulously prepared by removing water molecules, adding hydrogen atoms, and optimizing the structures using appropriate force fields. Candidate compounds identified from the TCMSP database underwent optimization for docking, with their 3D structures generated and geometries minimized using suitable algorithms and force fields. The prepared proteins and ligands were then subjected to molecular docking, wherein ligands were strategically placed into the binding sites of proteins, and their binding affinities were evaluated based on sophisticated scoring functions. Docking scores were computed to predict binding affinities, where lower scores indicated stronger predicted interactions. The docking results were subsequently validated through detailed visual inspection of binding poses.

**1.10 Light-Controlled Cascade Catalytic Reaction**

In a quartz cuvette, 2 mg of the photocatalyst was added to 2 mL of phosphate-buffer solution (PBS, 0.1 M, pH 7.0) containing varying concentrations of glucose. The suspension was continuously irradiated for 30 min. Subsequently, 200 μL of TMB solution (4 mM) and 1.8 mL of acetate buffer (0.1 M, pH 4.0) were added to the reaction mixture, followed by incubation in the dark for an additional 20 minutes. After filtration, the final reaction solution was analyzed using UV–vis spectroscopy.

**1.11 Peroxidase-Like Activity Mimic**

Referring to previous literature, 200 μL of TMB solution (4 mM) was added to 3.8 mL of acetate buffer (0.1 M, pH 4.0) containing 0.4 mg mL^–1^ catalyst and various concentrations of H_2_O_2_. After incubation in the dark for 20 min, the concentration-dependent blue color at 652 nm was recorded using UV–vis spectroscopy. The kinetic parameters were calculated according to the Michaelis–Menten equation:

$$V=V_{max}\left[ S \right] / (K_{m} + \left[ S \right])$$

where *V* and *V*_max_ were the initial and maximum reaction velocities, respectively, [*S*] represented the concentrations of substrate, and *K*_m_ represented Michaelis constant (the [*S*] at which the reaction velocity reached half of the maximum).

**1.12 Photoelectrochemical Measurements**

The Mott–Schottky (M–S) measurements were performed using a CHI660E electrochemical workstation (Chenhua Instruments, China) equipped with a standard three-electrode configuration. To prepare the catalyst ink, 5 mg of the active material was thoroughly ground with 2 mL of ethanol in an agate mortar to achieve uniform dispersion. The resulting slurry was then coated onto the conductive surface of indium tin oxide (ITO) glass (active area: 1 cm^2^) and dried at 60℃ for 12 h to obtain the working electrode. A graphite rod and an Ag/AgCl electrode served as the counter and reference electrodes, respectively. A 0.5 M Na_2_SO_4_ aqueous solution was used as the electrolyte.

The M–S measurements were conducted at frequencies of 500, 1000, and 1500 Hz with an amplitude of 0.01 V, scanning within a potential range of −1.2 V to +1.0 V (vs. Ag/AgCl). The measured potentials were converted to the normal hydrogen electrode (NHE) scale using the following equation:

$$E_{\mathrm{NHE}}= E_{Ag/AgCl}+0.059 pH+0.212$$

where *E*_NHE_ represented the potential relative to NHE, *E*_Ag/AgCl_ was the potential measured against the Ag/AgCl reference electrode, while the pH of the solution was maintained at 7.0 under 25℃.

**1.13 DFT Calculations**

First-principles density functional theory (DFT) calculations were performed with the Perdew-Burke-Ernzerhof (PBE) exchange-correlation functional in a plane wave pseudopotential implementation using the Vienna Ab-initio Simulation Packages (VASP). A cutoff energy of 500 eV for the plane-wave basis set was employed. The method of Methfessel-Paxton (MP) with a smearing width of 0.05 eV was adopted. Energy and force convergence criteria were set as 1 × 10^–5^ eV and 0.03 eV/Å, respectively. Vacuum layer with a thickness of 15 Å was used to avoid spurious interactions between adjacent image cells. Grimme’s DFT-D3 scheme was used to account for the van der Waals interactions throughout the calculations.

**1.14 Hemolysis Assay**

Fresh anticoagulated red blood cells were washed three times with physiological saline and resuspended to a 2% (v/v) solution. Equal volumes of the erythrocyte suspension were mixed with physiological saline (negative control), ultrapure water (positive control), or WCN_x_-Rh2 suspensions at concentrations of 25, 50, 100, 200, 400, and 800 μg mL^–1^. The mixtures were incubated at 37℃ for 1–2 h with gentle shaking to avoid mechanical hemolysis. After centrifugation at 1000 rpm for 10 min, the supernatant was collected, and absorbance at 540–545 nm was measured using a microplate reader. The hemolysis rate (%) was calculated as:

$$\text{Hemolysis rate (\%)}=\frac{A_{\text{sample}} -A_{\text{NC}}}{A_{\text{PC}} -A_{\text{NC}}} \times100\%$$

where *A*_sample_ represented the absorbance of the sample solution, *A*_NC_ denoted the absorbance of physiological saline, and *A*_PC_ corresponded to the absorbance of ultrapure water.

**1.15 CCK-8 Assay**

DCs were seeded in 96-well plates at 5 × 10^3^–1 × 10^4^ cells per well in complete medium (DMEM supplemented with 10% FBS and 1% penicillin-streptomycin) and allowed to adhere for 12–24 h at 37℃ in 5% CO_2_. Sterile WCN_x_-Rh2 suspensions were prepared and diluted in the same medium to final concentrations of 25, 50, 100, 200, 400, and 800 μg mL^–1^, while vehicle-only medium served as the control. The culture medium was then replaced with 100 μL of the corresponding formulations, and cells were incubated for 24, 48, or 72 h. Parallel wells containing material suspensions without cells were set as background controls for optical interference correction.

At the designated time points, 10 μL of CCK-8 reagent was added to each well (1:10, v/v) and incubated for 1–2 h at 37℃ protected from light. The absorbance at 450 nm was measured using a microplate reader. Cell viability (%) was calculated as

$$\text{Cell viability}(\%)=\frac{A_{\text{sample}}-A_{\text{blank}}}{A_{\text{control}}-A_{\text{blank}}}\times100\%,$$

where *A*_sample_ represented the absorbance of material-treated cells, *A*_control_ was that of the untreated control group, and *A*_blank_ corresponded to the blank wells (medium + CCK-8, without cells).

**1.16 Construction of an in Vitro High-Glucose Stress Model**

Glucose provides the carbonyl substrate for nonenzymatic glycation, while BSA provides amino groups necessary for the formation of AGE, and H_2_O_2_ promotes glyco-oxidation to mimic oxidative stress under hyperglycemic conditions. DCs were seeded in 24-well plates (2–3 × 10^5^ cells per well) and cultured overnight in RPMI-1640 medium (10% FBS, 1% penicillin-streptomycin). The BSA concentration was fixed at 1% (w/v), while glucose concentrations were set at 0, 10, 20, 30, 40, and 50 mM and H_2_O_2_ concentrations at 0, 5, 25, 50, 75, and 100 μM, forming an orthogonal experimental matrix. Cells were treated with the respective glucose–H_2_O_2_–BSA combinations for 24 h, and culture supernatants were collected and centrifuged at 10,000 rpm for 10 min at 4℃. The AGEs content in the supernatants was measured using a commercial AGEs assay kit.

**1.17 Measurement of Intracellular and Extra****cellular AGEs**

Five experimental groups were established: blank control, high-glucose model, high-glucose + VIS, WCN_x_ + VIS, and WCN_x_-Rh2 + VIS. VIS (0.1W cm^–2^) was applied only to the designated groups according to the experimental design. After treatment according to the experimental design, culture supernatants were collected for extracellular AGEs determination, while DCs were washed three times with PBS and lysed to assess intracellular AGEs. All samples were centrifuged at 10,000 rpm for 10 min at 4℃, and the supernatants were collected for analysis. AGE levels were quantified using a commercial AGEs assay kit.

**1.18 DCFH-DA Assay for Intracellular ROS Levels**

DCs were divided into five groups: blank control, high glucose model, high glucose + VIS, WCN_x_ + VIS, and WCN_x_-Rh2 + VIS. After corresponding treatments, cells were washed three times with PBS and incubated with 10 μM DCFH-DA diluted in serum-free medium at 37℃ for 30 min in the dark. Subsequently, cells were washed thoroughly with PBS to remove excess probe, resuspended in PBS, and analyzed by flow cytometry to quantitatively determine intracellular ROS levels.

**1.19 Flow Cytometry for DC Maturation**

DCs from five groups (blank control, high glucose model, high glucose + VIS, WCN_x_ + VIS, and WCN_x_-Rh2 + VIS) were collected and stained with fluorescein-labeled antibodies CD80-FITC and CD86-APC on ice for 30 min in the dark. After being washed twice with PBS, cells were resuspended and analyzed using a flow cytometer (Beckman Coulter, USA). Data were processed with FlowJo V10 software.

**1.20 Assessment of Intercellular Interactions**

*1.20.1 Measurement of HCECs Migration*

HCECs were seeded in 6-well plates and grown to 95–100% confluence; DCs (1–2 × 10^5^ cells per insert) were prepared in 0.4-µm Transwell inserts and assigned to five groups: blank control, high-glucose model, high-glucose + VIS, WCN_x_ + VIS, and WCN_x_-Rh2 + VIS. A linear scratch was made on the HCEC monolayer with a sterile 200-µL pipette tip, debris was removed by two PBS washes, and serum-reduced fresh medium was added. Inserts containing the pretreated DCs were then placed onto the scratched HCECs. Phase-contrast images were captured at 0 h, 24 h, and 48 h after co-culturation, and HCEC migration was quantified in ImageJ as wound closure:

$$\text{Migration (\%)}=\frac{A_{0} -A_{t}}{A_{0}} \times100\%$$

where *A*_0_ represented the initial wound area at 0 h, and *A*_t_ represented the wound area at time *t* after incubation.

*1.20.2 EdU Proliferation Assay*

HCECs were seeded on glass coverslips in 24-well plates (~5 × 10⁴ cells per well) and grown to ~70% confluence; DCs (1–2 × 10^5^ cells per insert) in 0.4-µm Transwell inserts were assigned to five groups (blank control, high glucose model, high glucose + VIS, WCN_x_ + VIS, and WCN_x_-Rh2 + VIS) and pretreated accordingly. Inserts were placed above HCECs for 24 h co-culture. Before EdU labeling, HCECs were washed twice with PBS (pH 7.4) to remove residual medium and serum components, then incubated with 10 µM EdU diluted in serum-free medium for 2 h at 37℃. Cells were then fixed with 4% paraformaldehyde for 15 min, permeabilized with 0.3% Triton X-100 for 10 min, reacted with the working solution for 30 min in the dark, counterstained with Hoechst, mounted, and imaged under a fluorescence microscope. Proliferation was quantified as the percentage of EdU⁺ nuclei among total Hoechst⁺ nuclei using ImageJ.

*1.20.3 Axon Staining*

Primary neurons were seeded on Poly-D-lysine–coated glass coverslips in 24-well plates (~4–6 × 10^4^ cells per well) and cultured to ~60–70% confluence. DCs (1–2 × 10^5^ cells per insert) in 0.4-µm Transwell inserts were divided into five groups: blank control, high-glucose model, high-glucose + VIS, WCN_x_ + VIS, and WCN_x_-Rh2 + VIS. Inserts were placed above neurons for 24 h of co-culture according to the group design. Cells were then fixed with 4% paraformaldehyde for 15 min, permeabilized with 0.1–0.3% Triton X-100 for 10 min, blocked with 5% BSA for 1 h, and incubated with anti-β3-tubulin antibody at 4℃ overnight. After washing, cells were incubated with Alexa Fluor–conjugated secondary antibody for 1 h at room temperature in dark, counterstained with Hoechst, and mounted. Fluorescence images were acquired under a fluorescence microscope, and axonal growth was analyzed using ImageJ to measure the longest axon length.

**1.21 WB**

Cells were lysed using RIPA buffer (Servicebio, China) supplemented with protease and phosphatase inhibitors. The protein concentration was then assessed with the BCA protein assay kit (Servicebio, China). The protein samples were denatured by adding a protein super sampling buffer and subsequently transferred to a PVDF membrane (Servicebio, China). The membrane was blocked with 5% skimmed milk for 1 h, followed by incubation with the primary antibody at 4℃ overnight. After washing away any residual antibody with Tris Buffered Saline containing Tween® 20 (TBST), a secondary antibody was added for 30 min. The membranes were then treated with a mixed ECL luminescent solution to visualize the immunoblot, and the results were analyzed using ImageJ software.

**1.22 Evaluation of in Vitro Antibacterial Performance**

The antibacterial activity of materials was assessed under two conditions: (i) different concentrations of the final material under 808 nm irradiation and (ii) different material components with or without 808 nm laser irradiation. *P. aeruginosa* and *S. aureus* were cultured overnight in LB medium, diluted to ~10^6^ CFU mL^–1^, and mixed with material suspensions of different groups in sterile centrifuge tubes. For the irradiation groups, samples were exposed to an 808 nm laser (1 W cm^–2^) intermittently—irradiated for 1 min at 1 min intervals, for a total irradiation duration of 10 min—while the dark groups were maintained under identical conditions without light. All samples were then incubated at 37℃ for 6 h with gentle shaking. After co-culture, 100 μL of each bacterial suspension was serially diluted, spread onto LB agar plates, and incubated at 37℃ overnight. Colony counts were recorded, and antibacterial efficiency was calculated as

$$\text{Inhibition rate (\%)}=\frac{N_{0} - N_{t}}{N_{0}} \times100\%$$

where *N*_0_ was the colony count of the control group, and *N*_t_ was that of the treated group.

**1.23 Bacterial Live/Dead Staining Assay**

Overnight cultures of bacteria were diluted to ~10^6^ CFU mL^–1^ and divided into four groups: control, control + 808 nm, WCN_x_-Rh2, and WCN_x_-Rh2 + 808 nm. Suspensions were mixed with PBS or WCN_x_-Rh2 as designed and incubated at 37℃ for 6 h. After treatment, the samples were centrifuged at 3000 rpm for 15 min, washed once with PBS, and resuspended. The bacterial suspensions were stained with DMAO/PI dye (Beyotime) and incubated for 15 min at room temperature in the dark. Stained samples were mounted on slides and imaged using a confocal laser scanning microscope (60× objective) with excitation/emission settings of 488/500–550 nm for DMAO (live, green) and 561/600–650 nm for PI (dead, red). ImageJ software was used to calculate the proportion of live and dead bacteria.

**1.24 BCA Protein Leakage Assay**

Bacterial suspensions were adjusted to 10^6^–10^7^ CFU mL^–1^ in PBS and divided into two groups: control and WCN_x_-Rh2 + 808 nm. The light group received 808 nm laser irradiation (1 W cm⁻^2^, 1 min on / 1 min off, total 10 min), while the control group was kept in the dark. After irradiation, all samples were co-cultured at 37℃ for 6 h with gentle shaking. The suspensions were then centrifuged at 3000 rpm for 15 min, and the supernatants were collected for protein quantification using a BCA protein assay kit according to the manufacturer’s instructions. BSA standards were prepared, and each sample (or its dilution) was mixed with BCA working reagent, incubated at 37℃ for 30 min, and measured at 562 nm using a microplate reader. The leaked protein content was calculated based on the BSA standard curve.

**1.25** **Crystal Violet Biofilm Assay**

Bacterial suspensions were adjusted to ~10^7^ CFU mL^–1^ in LB, and 500 μL of bacterial suspension was added to each well of a sterile, flat-bottom 48-well plate. Plates were incubated statically at 37℃ for 72 h to allow biofilm formation, then wells were divided into four groups: control, control + 808 nm, WCN_x_-Rh2, and WCN_x_-Rh2 + 808 nm (WCNx-Rh2 was added to the designated wells at the intended working concentration). Light groups were irradiated with an 808 nm laser (1 W cm⁻^2^, 1 min on / 1 min off, total 10 min), while dark groups were kept unexposed. After treatment, planktonic cells were discarded; wells were gently washed 3× with PBS (500 μL each), fixed with methanol (500 μL, 15 min), stained with 0.1% (w/v) crystal violet (500 μL, 15 min), rinsed thoroughly with water, and air-dried. The stained biofilms were photographed for visual documentation. Finally, 33% acetic acid (500 μL, 15 min) was added to dissolve the bound dye, and absorbance at 590 nm was measured using a microplate reader. Biofilm inhibition was expressed as relative OD_590_ versus the blank group.

**1.26 Induction of Diabetes Mellitus in Mice**

Male C57BL/6J mice (6–8 weeks) were obtained from Beijing Vital River Laboratory Animal Technology Co., Ltd. (Beijing, China) and housed under specific pathogen–free (SPF) conditions (22–24℃, 12 h light/dark cycle, free access to food and water). All animal procedures conformed to the *Guidelines for the Care and Use of Laboratory Animals*, the *Principles for the Utilization and Care of Vertebrate Animals*, and the ARVO Statement for the Use of Animals in Ophthalmic and Vision Research. The experimental protocol was approved by the Institutional Animal Care and Use Committee of Nanchang University (IACUC approval number: NCULAE-20250901007). Streptozotocin (STZ; Sigma-Aldrich, USA) was freshly dissolved in 0.1 M citrate–citric acid buffer (pH 4.5) immediately before injection. Experimental mice received intraperitoneal injections of low-dose STZ (50 mg kg^–1^ day^–1^) for five consecutive days, while control mice were injected with an equal volume of citrate buffer. Baseline tail-vein blood glucose levels were measured prior to injection, and random blood glucose levels were monitored every 4 weeks until week 16. All mice were fed a standard chow diet. Mice with blood glucose levels exceeding 16.7 mM in each measurement were identified as diabetic.

**1.27 Measurement of Corneal Sensitivity**

Corneal mechanical sensitivity was assessed using a Cochet–Bonnet esthesiometer (Luneau Ophthalmology, France). Unanesthetized mice were tested every 4 weeks (weeks 4, 8, 12, and 16) during the diabetes protocol; an additional measurement was performed on day 5 after corneal epithelial debridement. For each session, both eyes were tested ≥ 3 times. The nylon filament was initially set to maximal length and progressively shortened to 0.5-cm steps until a positive blink reflex was elicited. The corneal sensitivity threshold was defined as the longest filament length producing a positive response.

**1.28 Whole-Mount Immunofluorescence Staining of Mouse Cornea**

Mice were euthanized on day 5 after corneal epithelial debridement, intact corneas were dissected, rinsed in PBS, and fixed in Zamboni’s fixative (RT, 30–60 min). After three PBS washes, each cornea was radially cut into a four-leaf clover shape and incubated in blocking/permeabilization buffer (0.3% Triton X-100 + 10% normal goat serum) for 1 h at RT. Tissues were then incubated overnight at 4℃ with primary antibodies diluted in the same buffer: anti-β3-tubulin and anti-CD11c. After PBS washes (3 × 10 min), appropriate Alexa Fluor–conjugated secondary antibodies were applied for 1 h at RT in the dark. Nuclei were counterstained with Hoechst 33342 (1–5 min), rinsed, and corneas were flat-mounted epithelium-up with antifade medium. Whole mounts were imaged and captured on a laser confocal microscope.

**1.29 Elisa Assay**

Corneal tissues were collected 48 h after corneal epithelial debridement from diabetic and control mice. For each biological replicate, three corneas per group were pooled and homogenized in ice-cold PBS (10–20 μL per mg tissue) containing protease inhibitors. Homogenates were clarified (12,000 rpm, 10 min, 4℃), and total protein was quantified by BCA assay (Beyotime, China). Levels of NLRP3, GSDMD, IL-1β, and TNF-α were determined using mouse-specific commercial ELISA kits according to the manufacturers’ protocols and read on a microplate reader.

**1.30 Untargeted Metabolomic Analysis**

Metabolomic profiling of the normal control group, diabetic control group and WCN_x_-Rh2 + VIS treatment group was performed by APTBIO Co., Ltd. (Shanghai, China) using LC–MS/MS. The experimental protocol followed previously established procedures. Briefly, corneal tissue samples from different groups were collected and ground in liquid nitrogen, followed by extraction with 1 mL of pre-chilled methanol/acetonitrile/water (2:2:1, v/v). The mixtures were sonicated in an ice bath for 30 min, repeated once, then incubated at –20℃ for 1 h and centrifuged at 12,000 rpm for 20 min at 4℃. An aliquot of 200 µL supernatant was subjected to LC–MS/MS analysis. Principal component analysis (PCA) and orthogonal partial least squares-discriminant analysis (OPLS–DA) were performed to evaluate overall metabolic differences among groups. The KEGG database was used for metabolic pathway enrichment analysis.

**1.31 Establishment and Treatment of a Diabetic Corneal Keratitis Model**

After completion of diabetes induction, corneal keratitis was established by topical inoculation of *P. aeruginosa* (~1×10^8^ CFU mL^–1^, 5–10 μL per eye) following corneal epithelial debridement. Prior to surgery, mice were systemically anesthetized by intraperitoneal injection of Sumianxin II (Shutai, China), followed by topical ocular anesthesia using 0.5% proparacaine hydrochloride eye drops. A 2.0 mm corneal area was marked with a trephine, and the epithelium within the ring was gently removed using a sterile corneal scraper. Immediately after epithelial removal, a *P. aeruginosa* suspension was instilled onto the corneal surface (5–10 μL per eye), and the eyelids were gently closed for 30 s to facilitate bacterial adhesion. Mice were then randomly assigned to five groups: non-diabetic control (normal control), diabetic control, diabetic control + 808 nm, WCN_x_ + 808 nm, and WCN_x_-Rh2 + 808 nm. Treatments were performed once daily for seven consecutive days: the normal control, diabetic control, and diabetic control + 808 nm groups received PBS only; the diabetic control + 808 nm group received extra 808 nm laser irradiation; and the WCN_x_ + 808 nm and WCN_x_-Rh2 + 808 nm groups received topical instillation of the respective material (5–10 μL per eye) followed by 808 nm irradiation (0.8 W cm⁻^2^, 30 s on / 30 s off for total 3 min). During the treatment period, corneal conditions were evaluated daily using a slit-lamp microscope, assessing corneal opacity and edema under white light and epithelial wound healing with fluorescein sodium staining under cobalt-blue illumination. On day 7, mice were euthanized, and eyeballs were enucleated, fixed in 4% paraformaldehyde, paraffin-embedded, and sectioned for H&E, Gram, and Masson’s trichrome staining to analyze corneal inflammation, bacterial colonization, and collagen remodeling.

**1.32 Statistical Analysis**

Statistical analyses were performed using GraphPad Prism v.10.0 (GraphPad Software, Inc., San Diego, CA, USA). Data were presented as mean ± SD. Differences between groups were analyzed using an unpaired Student’s *t*-test or one-way analysis of variance (ANOVA). A *p*-value < 0.05 was considered statistically significant.


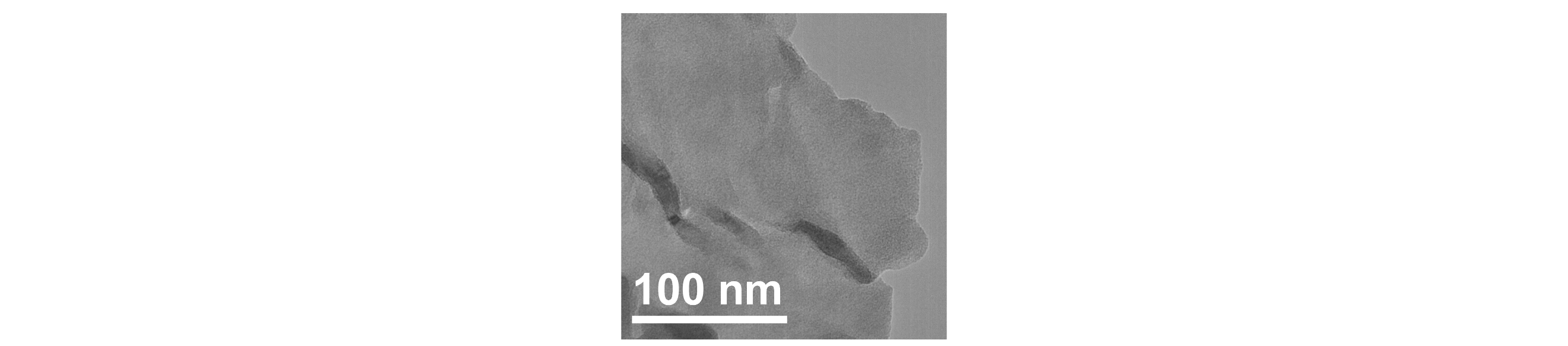


**Figure S1.** TEM image of CN.


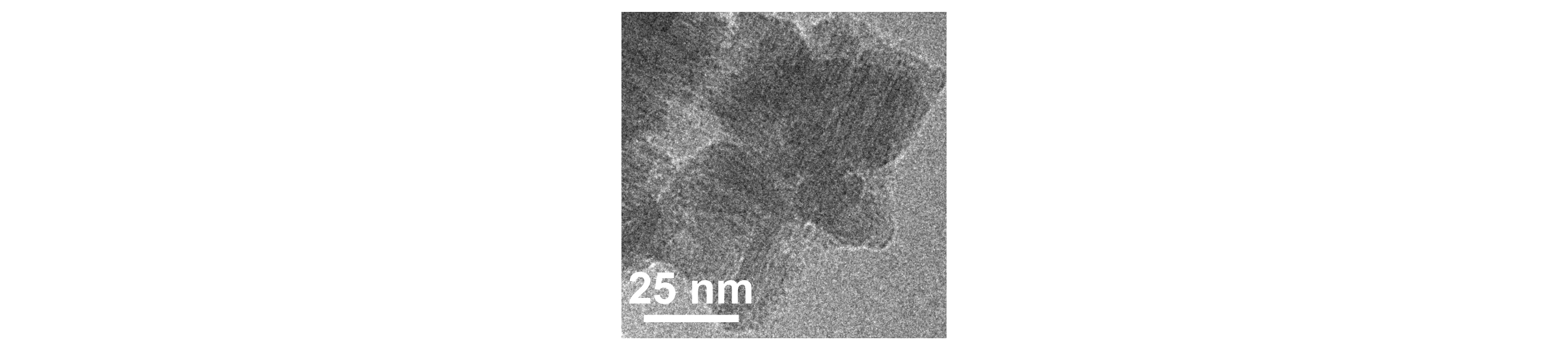


**Figure S2.** TEM image of WO_x_.


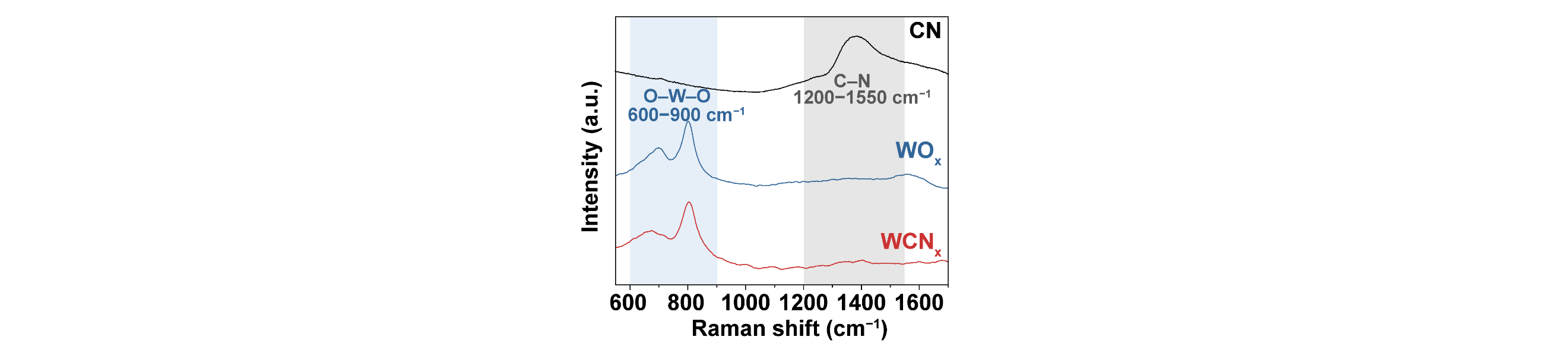


**Figure S3.** Raman spectra of CN, WO_x_, and WCN_x_.


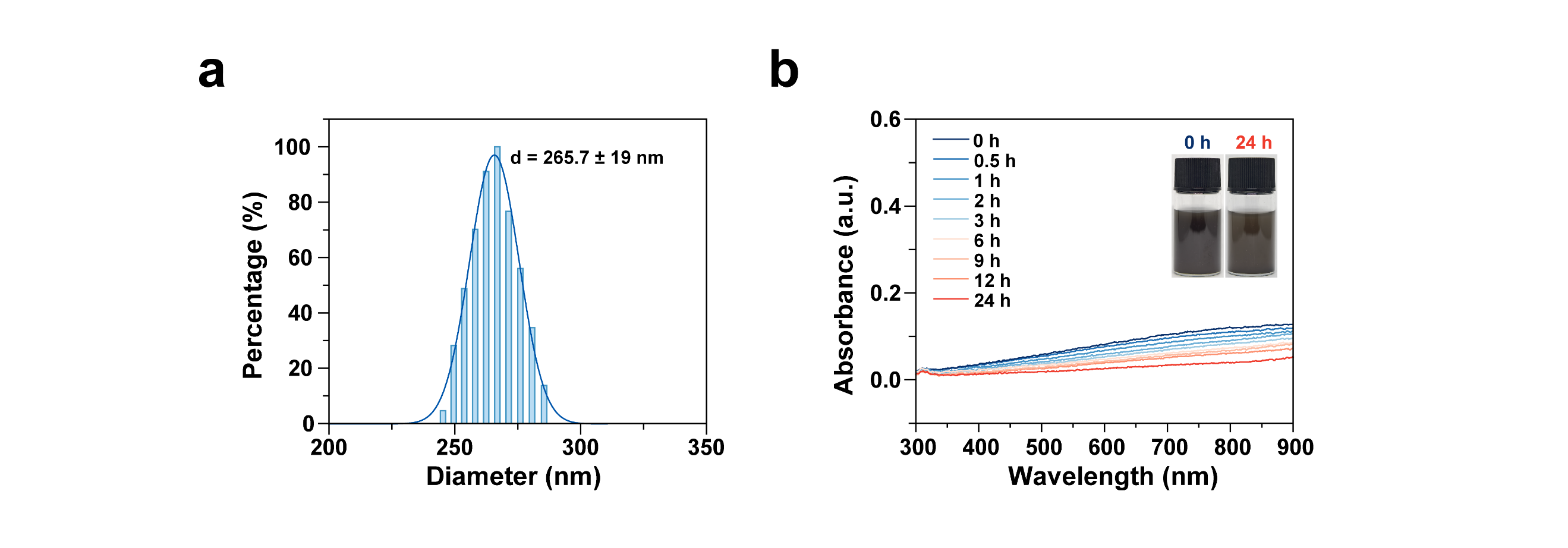


**Figure S4.** a) Particle-size analysis of WCN_x_. b) Evaluation of the dispersion stability of WCN_x_ in solution.


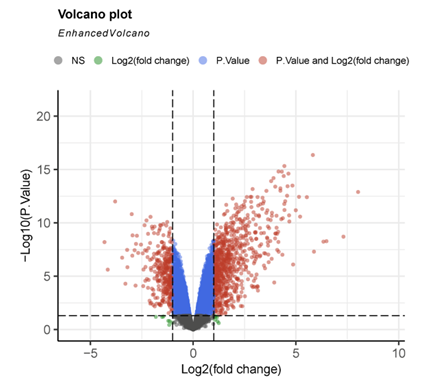


**Figure S5.** Volcano plots of differentially expressed genes in corneal tissues of normal and diabetic mice.


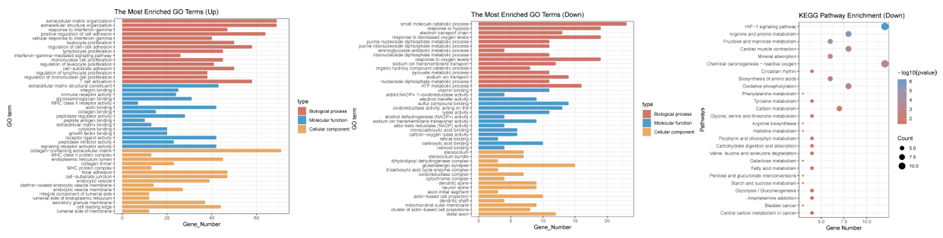


**Figure S6.** GO and KEGG enrichment analyses based on transcriptomic results.


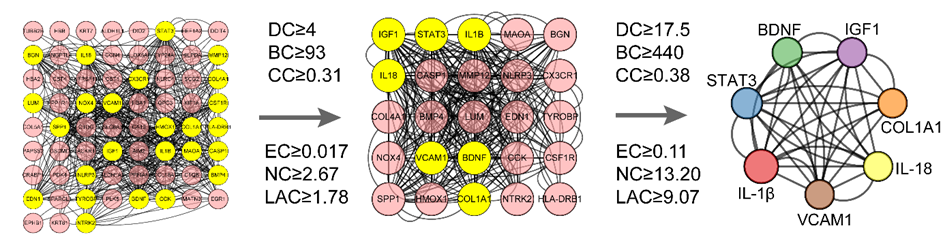


**Figure S7.** Identification of key genes from the PPI network using Cytoscape.


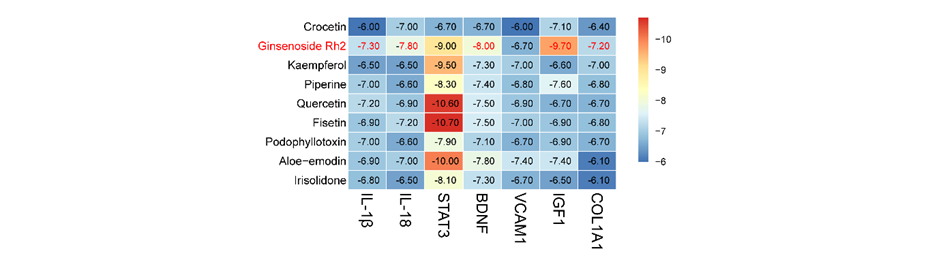


**Figure S8.** Target–compound–herb network.


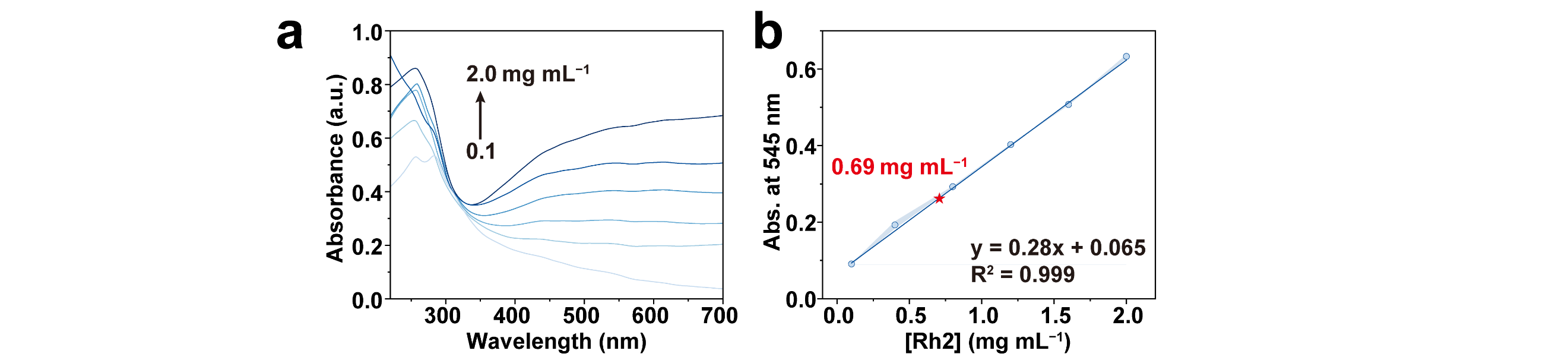


**Figure S9.** a) Standard calibration curve of Rh2 concentration and b) drug-loading capacity. Data are means ± SD (n ≥ 3).


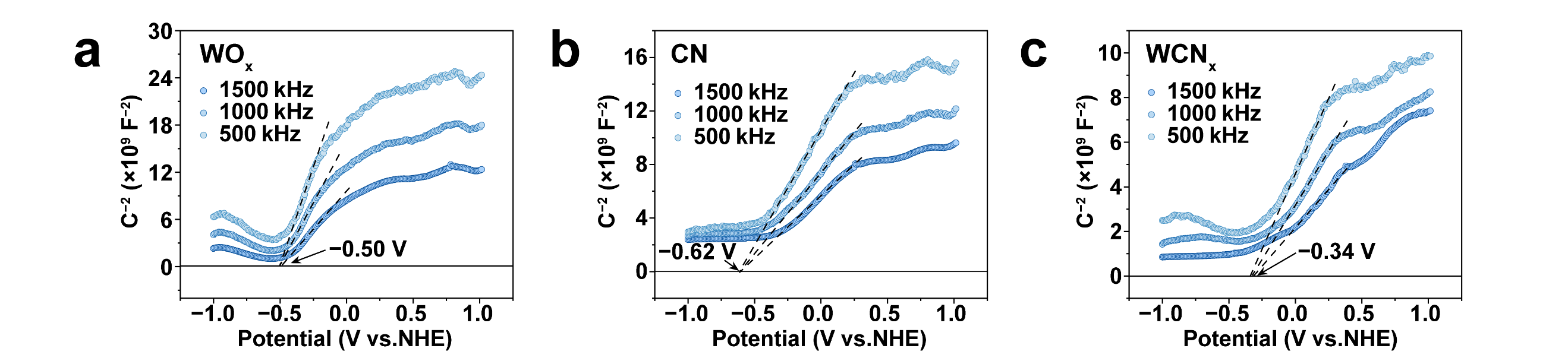


**Figure S10.** Mott–Schottky plots of a) CN, b) WO_x_, and c) WCN_x_.


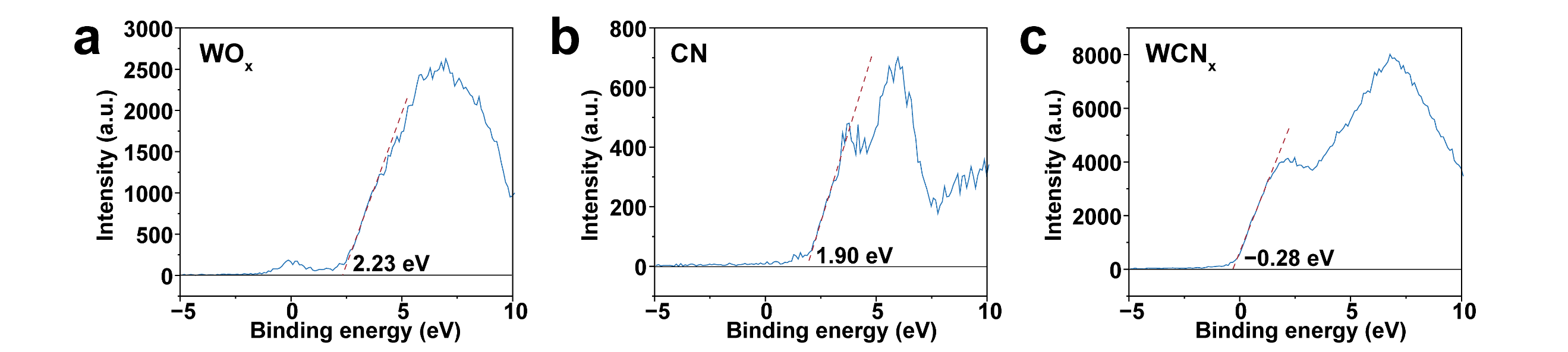


**Figure S11.** XPS valence-band spectra of a) CN, b) WO_x_, and c) WCN_x_.


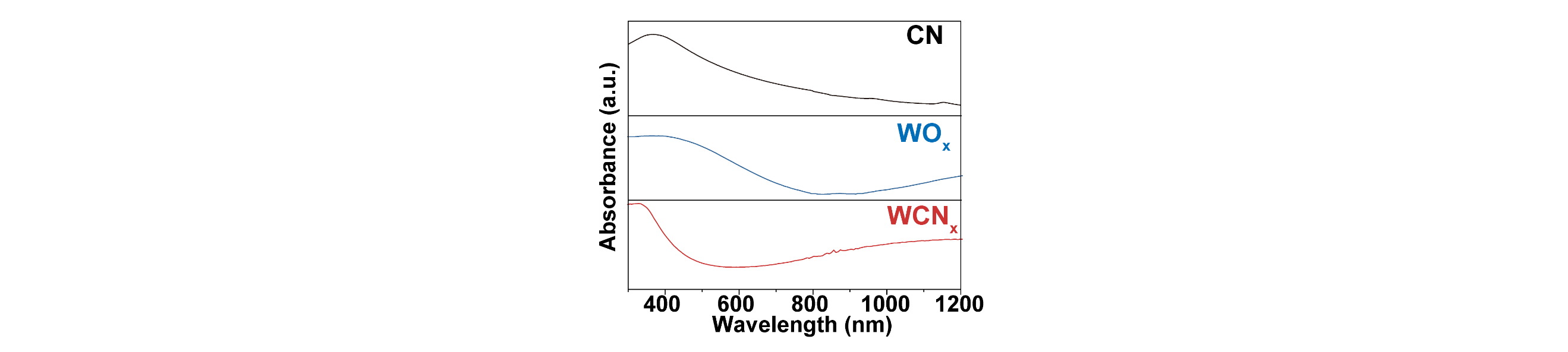


**Figure S12.** UV–vis–IR spectra of CN, WO_x_, and WCN_x_.


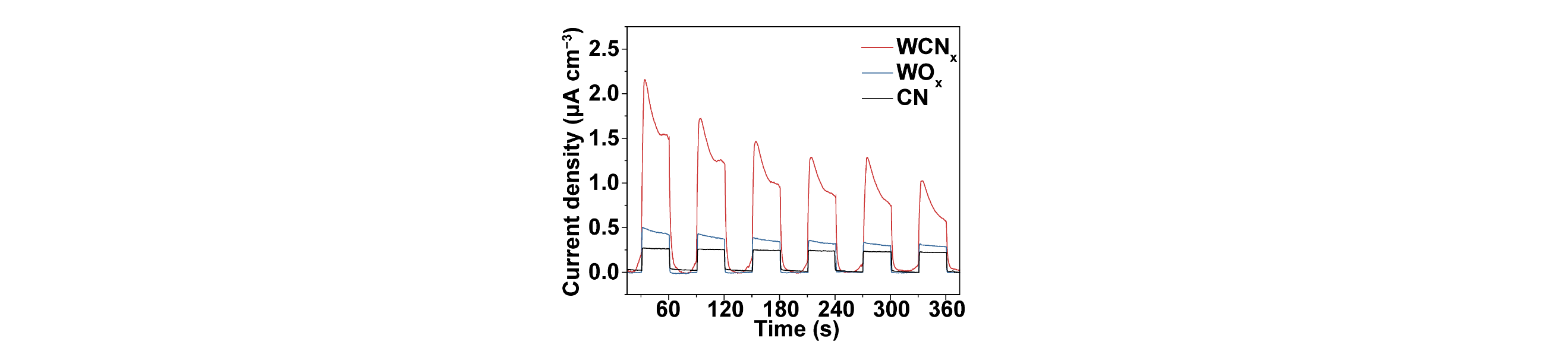


**Figure S13.** Photocurrent response curves of CN, WO_x_, and WCN_x_.


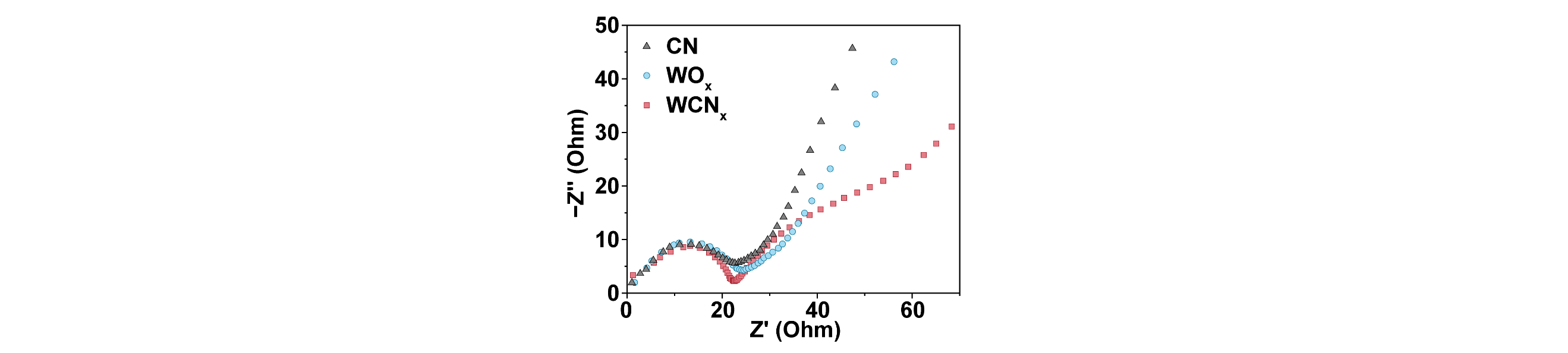


**Figure S14.** Electrochemical impedance spectra of CN, WO_x_, and WCN_x_.


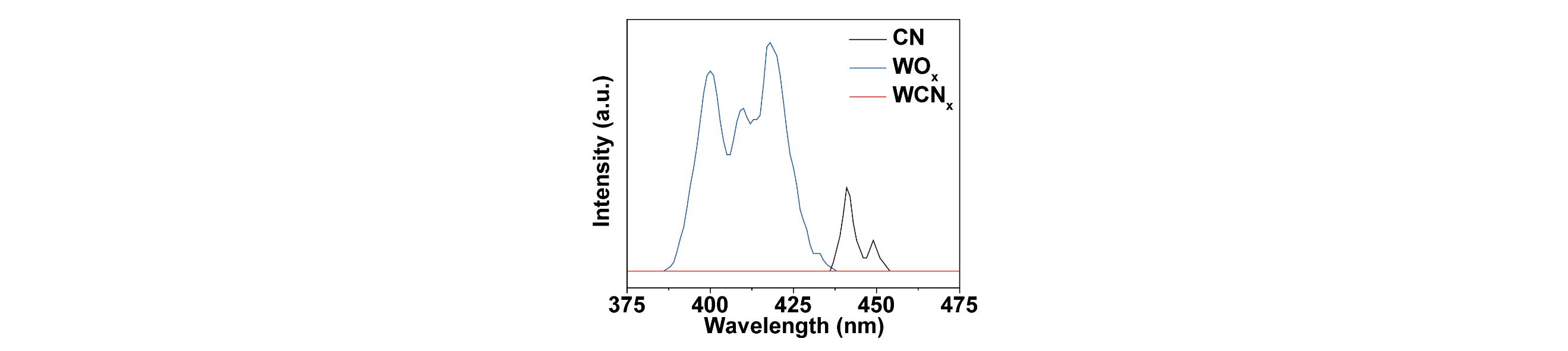


**Figure S15.** PL spectra of CN, WO_x_, and WCN_x_.


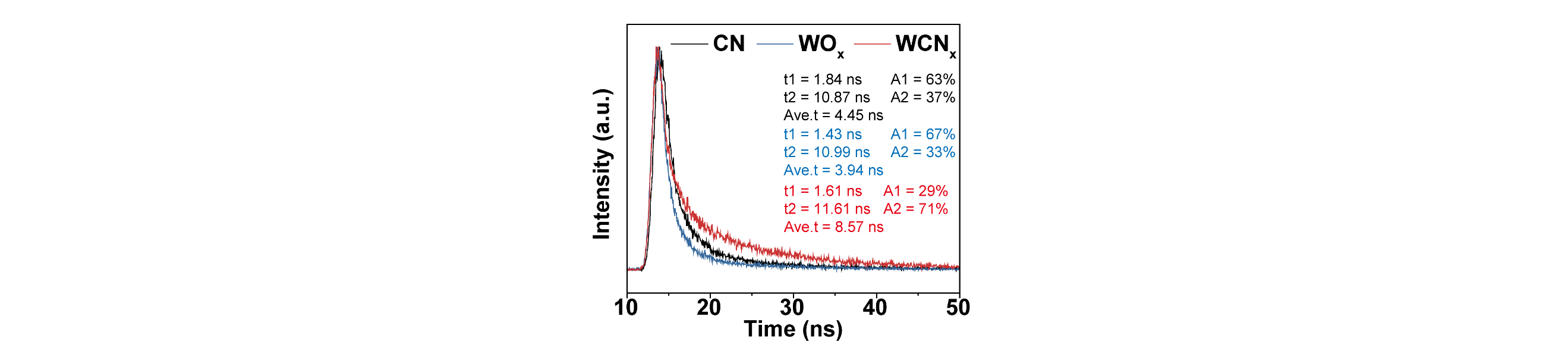


**Figure S16.** Transient and steady-state photoluminescence spectra of CN, WO_x_, and WCN_x_.


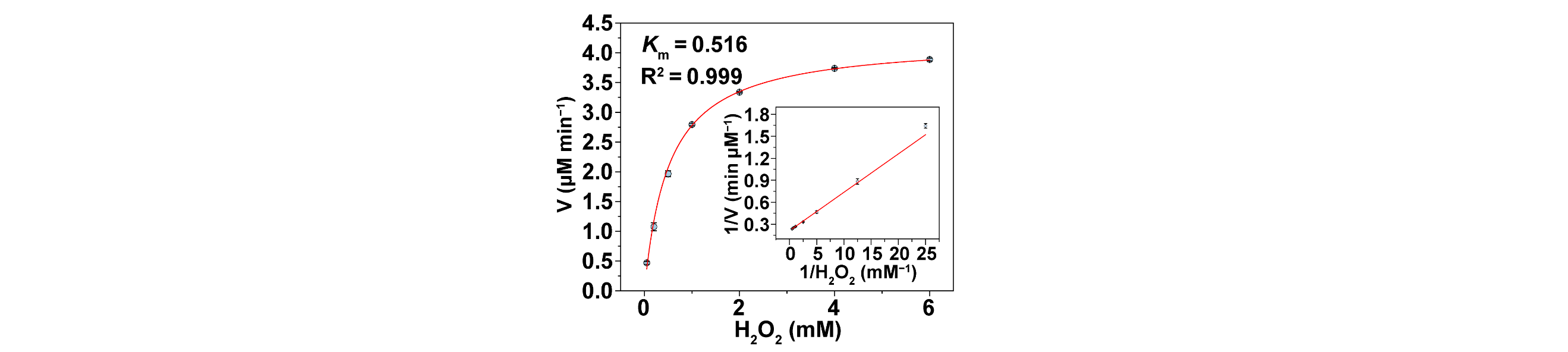


**Figure S17.** Kinetic fitting curves of the POD-like catalytic activity of WCN_x_. Data are means ± SD (n ≥ 3).


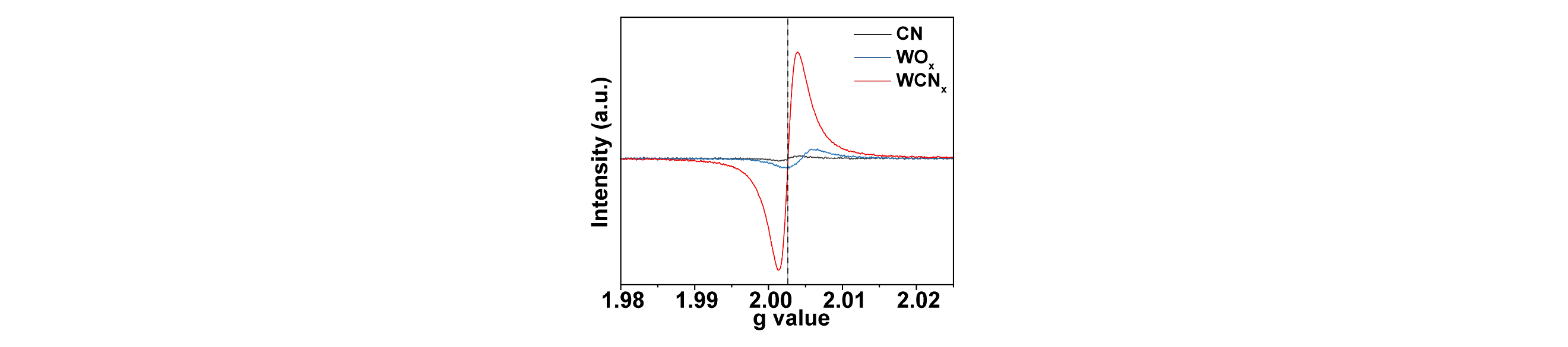


**Figure S18.** Electron paramagnetic resonance spectra of CN, WO_x_, and WCN_x_.


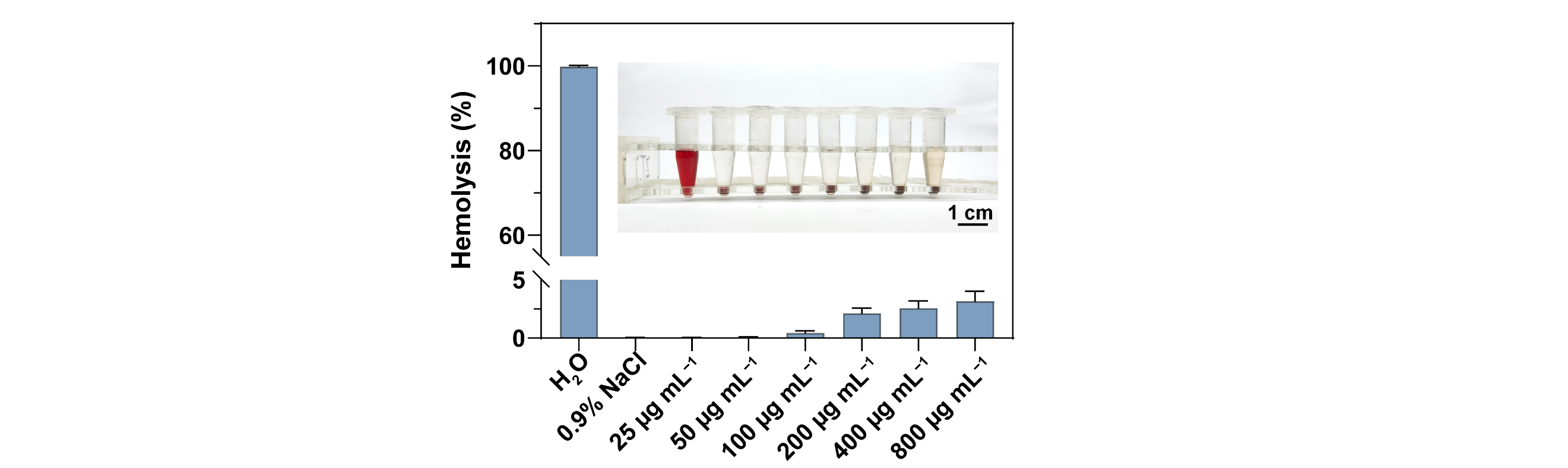


**Figure S19.** Hemolysis assay of WCN_x_-Rh2 with various concentrations. Data are means ± SD (n ≥ 3).


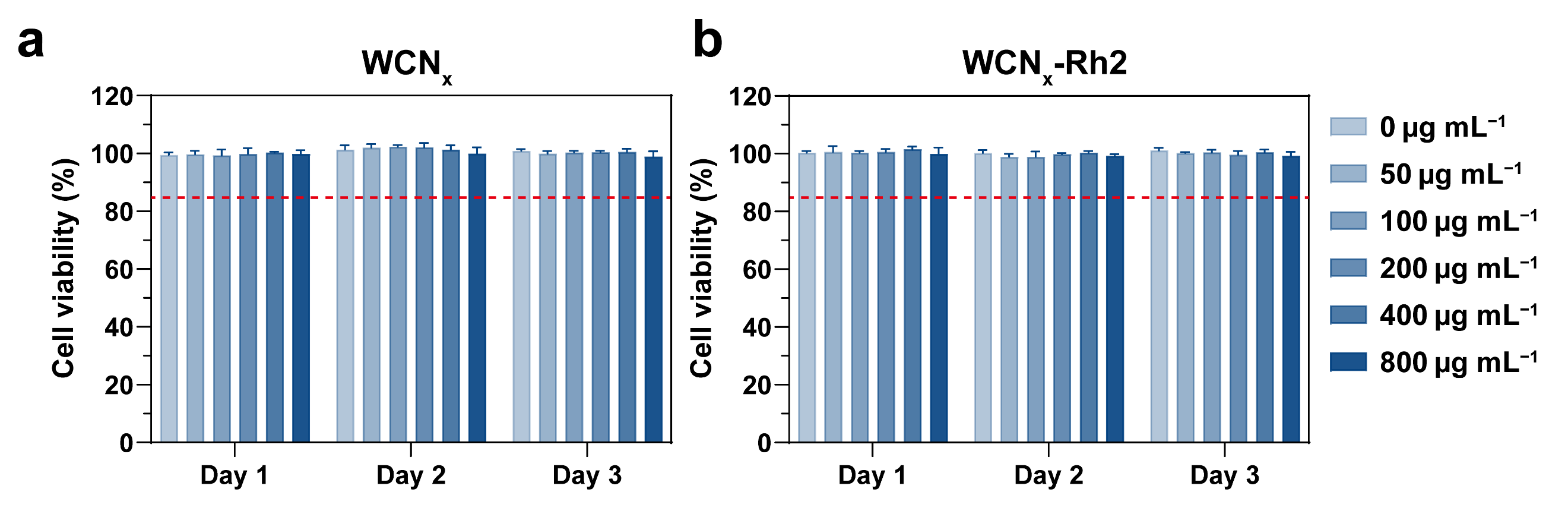


**Figure S20.** CCK-8 viability of a) WCN_x_ and b) WCN_x_-Rh2 at different concentrations on Days 1–3. Data are means ± SD (n ≥ 3).


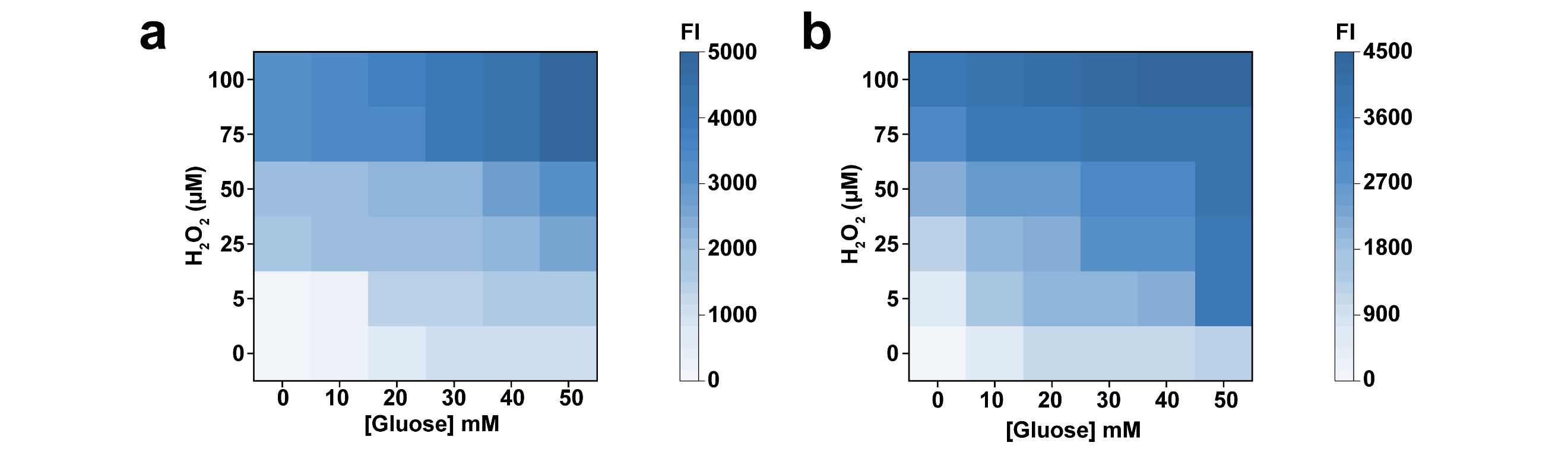


**Figure S21.** Levels of advanced glycation end-products under different glucose and H_2_O_2_ models in a) WCN_x_-treated groups and b) blank control groups. Data are means ± SD (n ≥ 3).


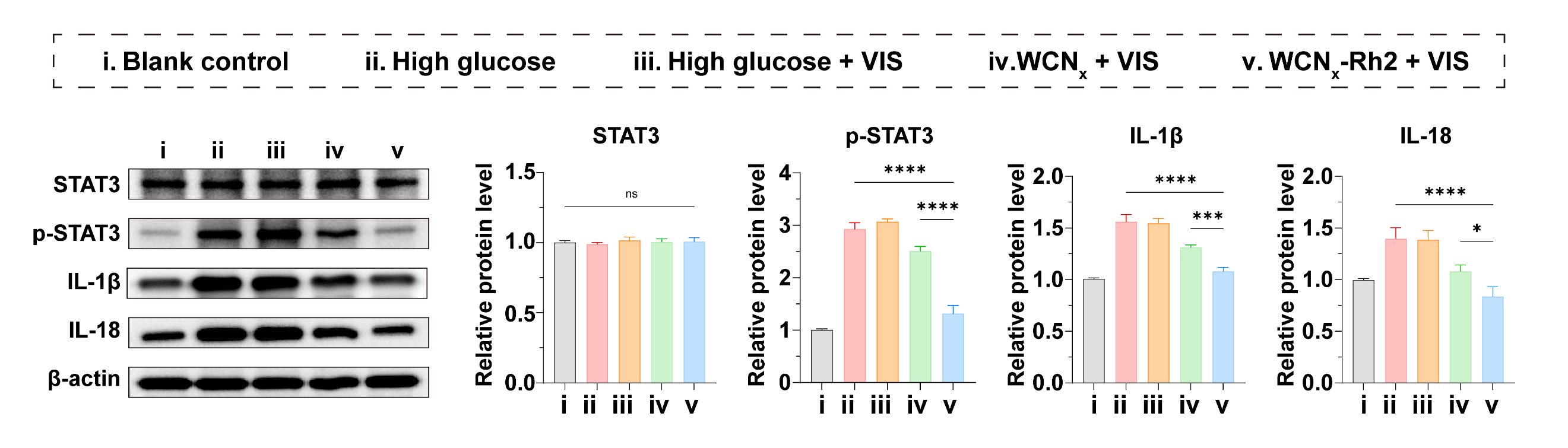


**Figure S22.** WB bands and quantitative analysis of STAT3 signaling pathway-related proteins. Data are means ± SD (n ≥ 3). ns = not significant, **p* < 0.05, ****p* < 0.001, *****p* < 0.0001.


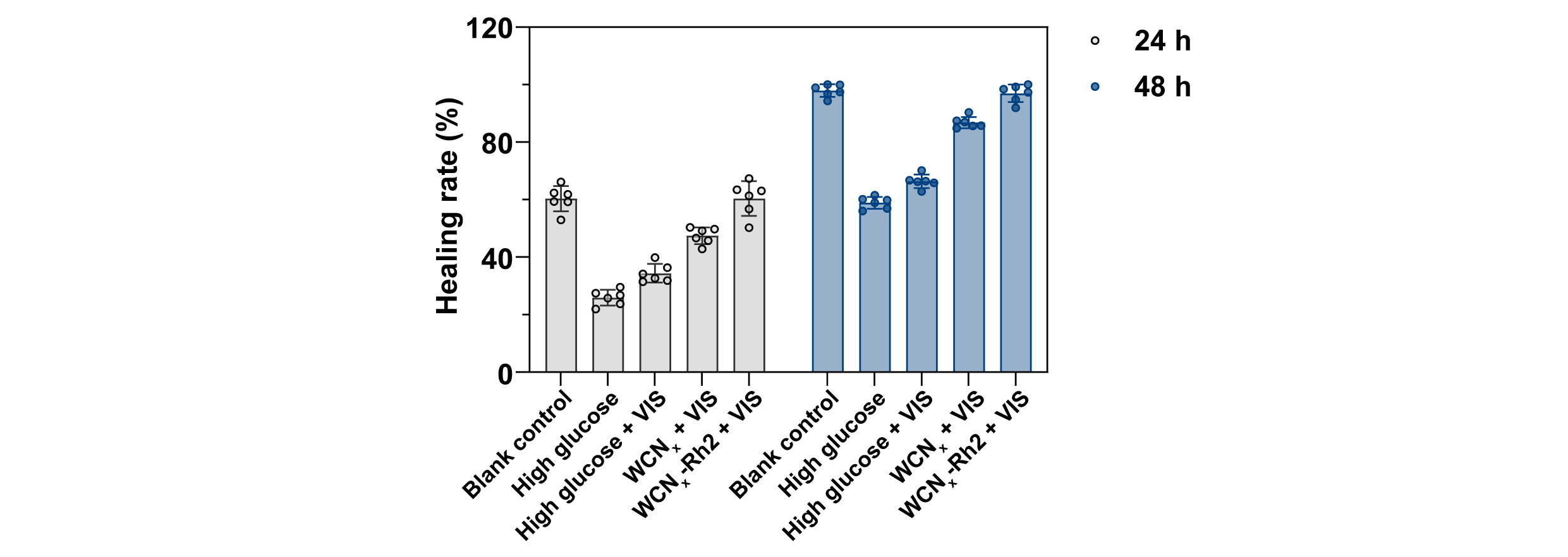


**Figure S23.** Quantification of the HCEC migration area. Data are means ± SD (n = 6).


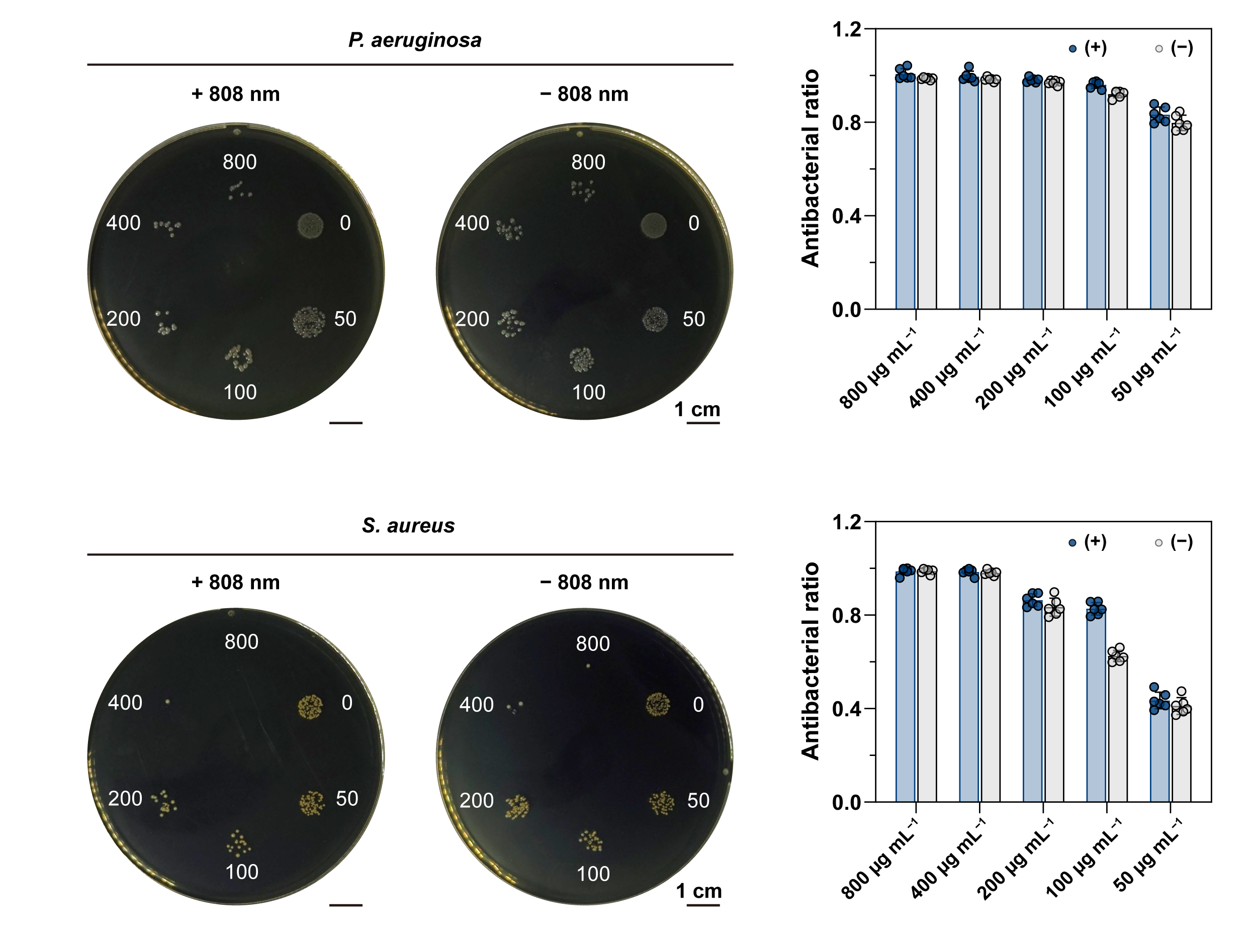


**Figure S24.** Antibacterial performance of WCN_x_ at different concentrations with and without 808 nm irradiation. Data are means ± SD (n = 6).


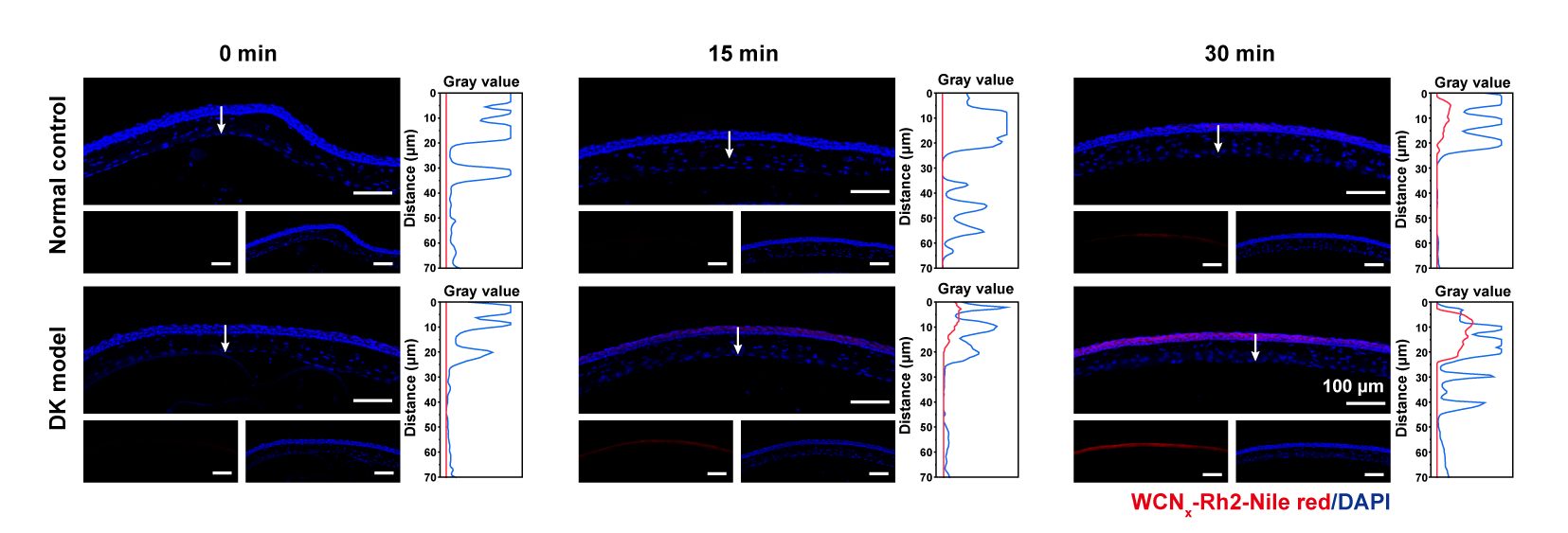


**Figure S25.** Representative fluorescence confocal images of ocular sections at 0, 15, and 30 min after topical instillation of Nile red-labeled WCN_x_-Rh2. The gray-value profiles on the right show the Nile red signal (red) and DAPI signal (blue).


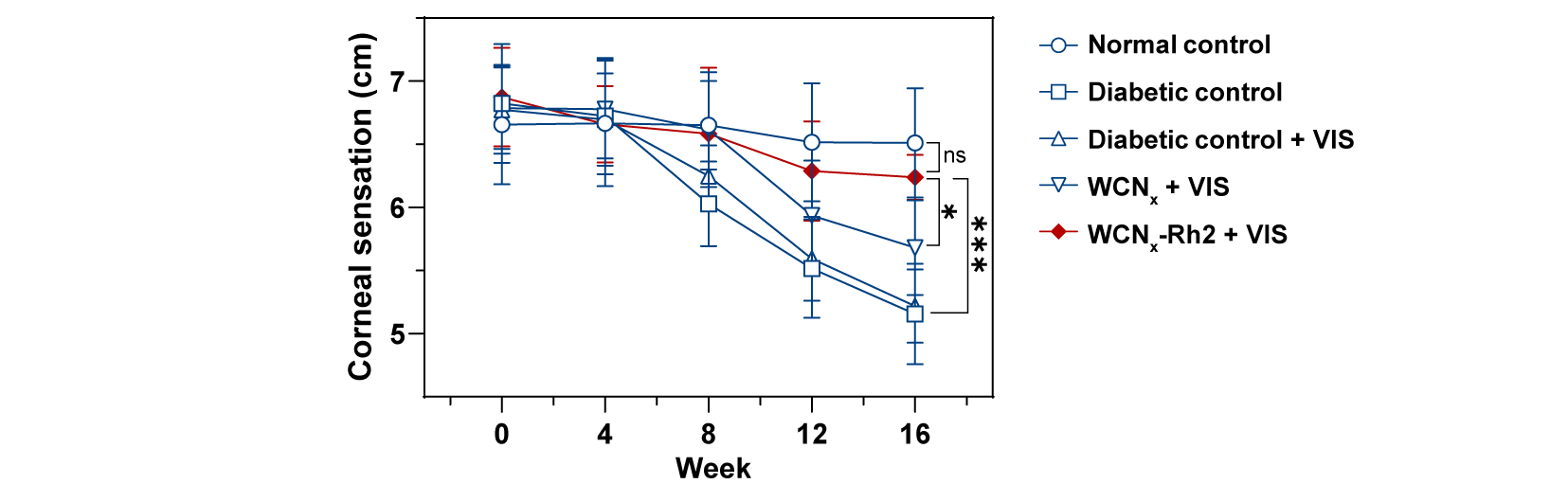


**Figure S26.** Corneal sensitivity testing in mice among different groups after establishment of the diabetic model. Data are means ± SD (n ≥ 3). ns = not significant, ****p* < 0.001.


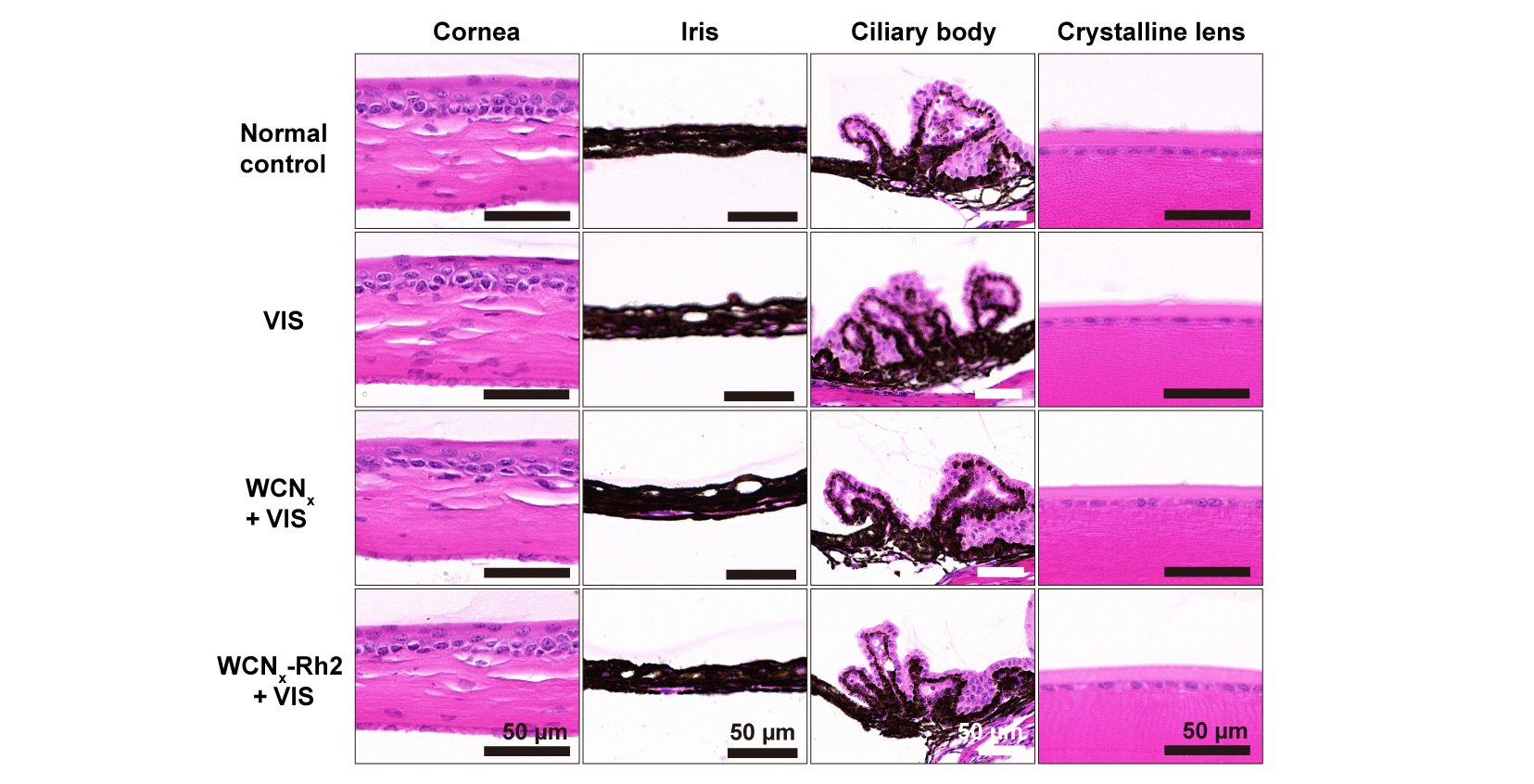


**Figure S27.** H&E staining of the cornea, iris, ciliary body, and crystalline lens in ocular tissues from different groups of mice.


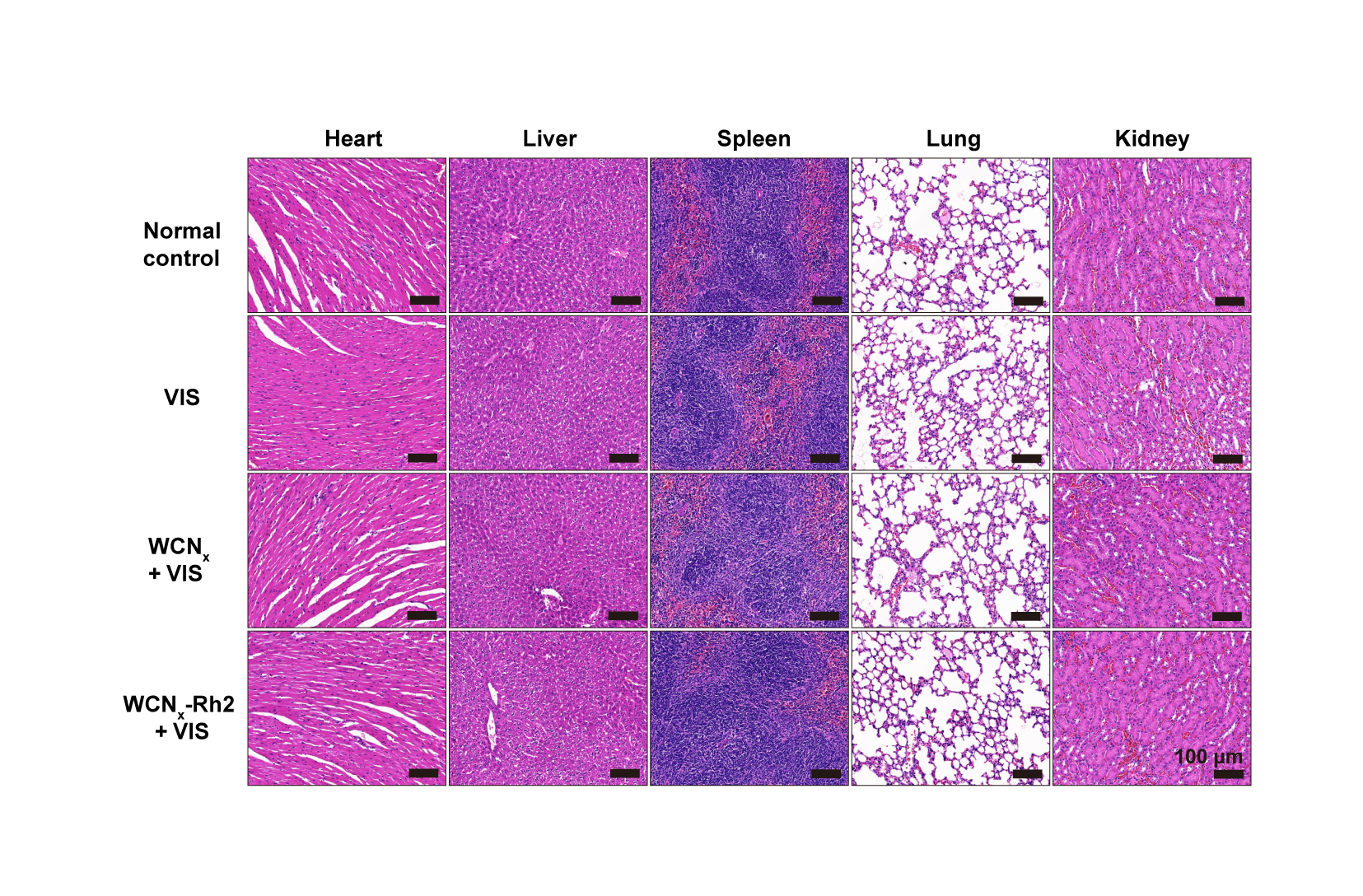


**Figure S28.** H&E staining of the heart, liver, spleen, lung, and kidney from different groups of mice.


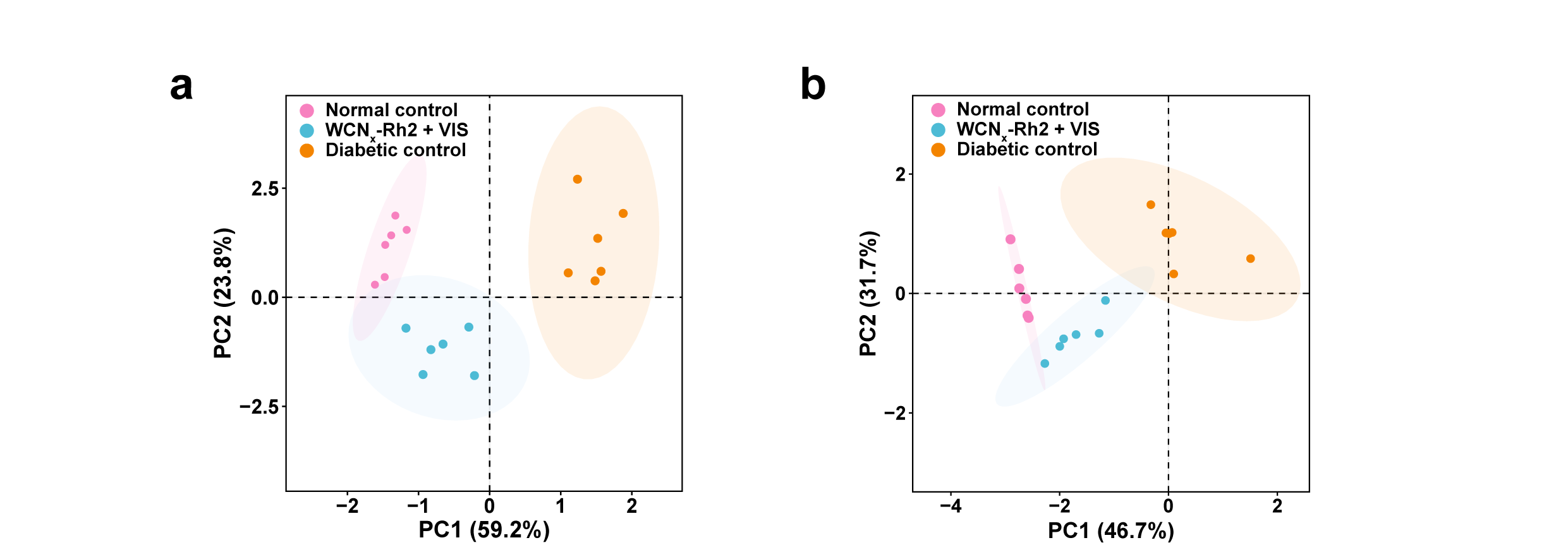


**Figure S29.** PCA score plots among different groups in a) the positive pattern and b) the negative pattern. (n = 6).


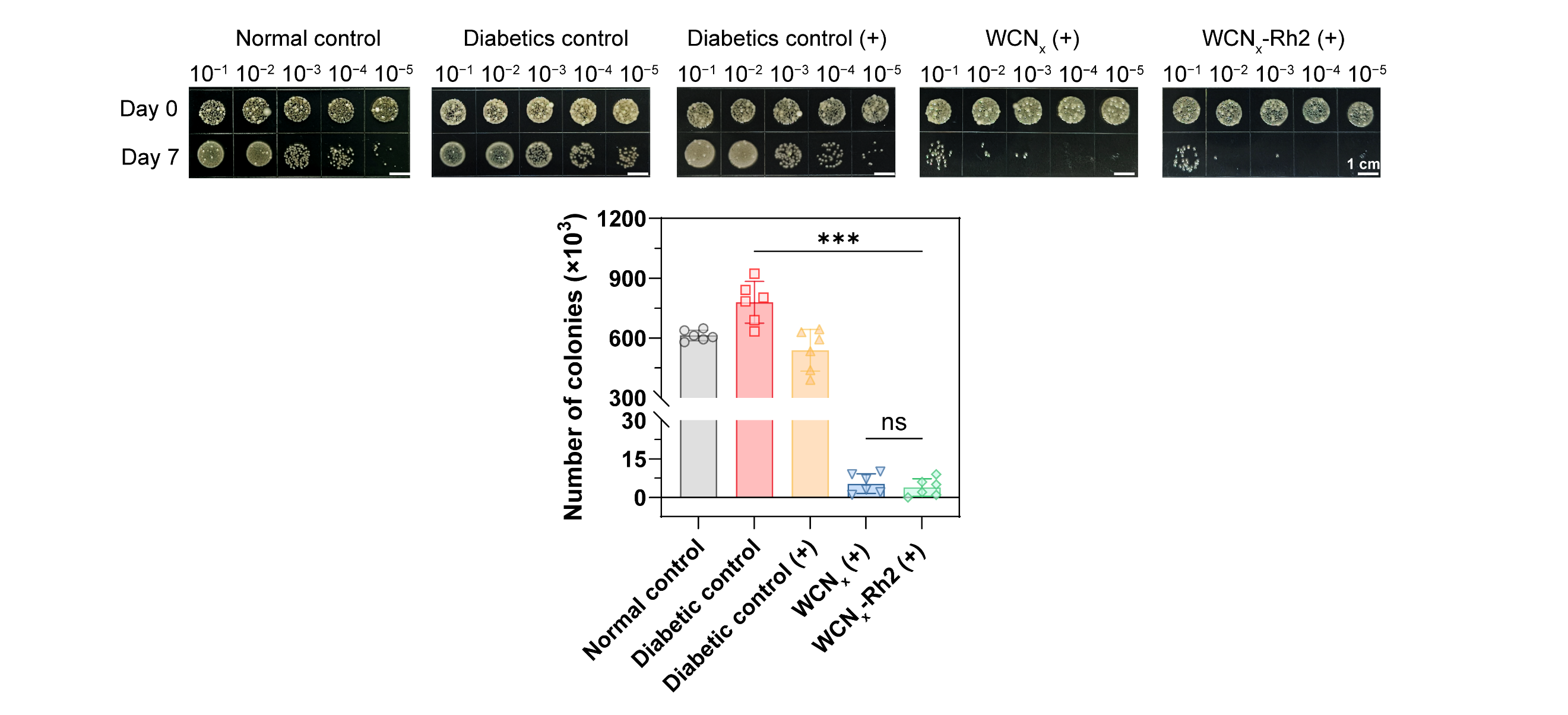


**Figure S30.** Representative plate culture images and quantitative colony count from ocular surface samples of different treatment groups on Day 0 and Day 7 in the diabetic keratitis model. Data are means ± SD (n = 6). ns = not significant, ****p* < 0.001.


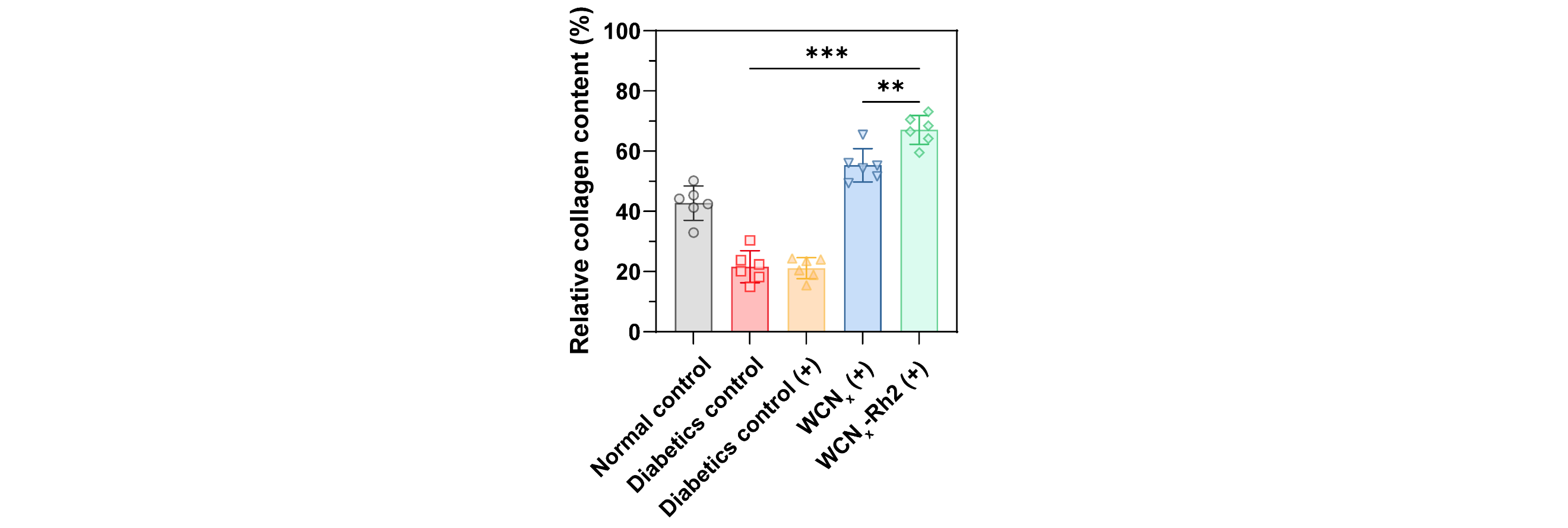


**Figure S31.** Relative collagen content in corneas of mice from different treatment groups. Data are means ± SD (n = 6). ***p* < 0.01, ****p* < 0.001.
